# Red piranha optimization (RPO): a natural inspired meta-heuristic algorithm for solving complex optimization problems

**DOI:** 10.1007/s12652-023-04573-1

**Published:** 2023-03-17

**Authors:** Asmaa H. Rabie, Ahmed I. Saleh, Nehal A. Mansour

**Affiliations:** Computers and Control Department, Faculty of Engineering Mansoura University, Mansoura, Egypt

**Keywords:** Optimization, Bio-inspired, Meta-heuristic, Piranha, Algorithms

## Abstract

An optimization algorithm is a step-by-step procedure which aims to achieve an optimum value (maximum or minimum) of an objective function. Several natural inspired meta-heuristic algorithms have been inspired to solve complex optimization problems by utilizing the potential advantages of swarm intelligence. In this paper, a new nature-inspired optimization algorithm which mimics the social hunting behavior of Red Piranha is developed, which is called Red Piranha Optimization (RPO). Although the piranha fish is famous for its extreme ferocity and thirst for blood, it sets the best examples of cooperation and organized teamwork, especially in the case of hunting or saving their eggs. The proposed RPO is established through three sequential phases, namely; (i) searching for a prey, (ii) encircling the prey, and (iii) attacking the prey. A mathematical model is provided for each phase of the proposed algorithm. RPO has salient properties such as; (i) it is very simple and easy to implement, (ii) it has a perfect ability to bypass local optima, and (iii) it can be employed for solving complex optimization problems covering different disciplines. To ensure the efficiency of the proposed RPO, it has been applied in feature selection, which is one of the important steps in solving the classification problem. Hence, recent bio-inspired optimization algorithms as well as the proposed RPO have been employed for selecting the most important features for diagnosing Covid-19. Experimental results have proven the effectiveness of the proposed RPO as it outperforms the recent bio-inspired optimization techniques according to accuracy, execution time, micro average precision, micro average recall, macro average precision, macro average recall, and f-measure calculations.

## Introduction

Recently, a wide spectrum of Nature-Inspired Optimization (NIO), also called meta-heuristic, techniques have considerably emerged to address real-world complex optimization problems by mimicking the natural phenomenon (Sharma and Kaur 2021; Monga et al. 2022). Some of them have proved advantage over conventional optimization algorithms, while the others are still under development. NIO, which are also named as meta-heuristic algorithms, have gained higher popularity in the last two decades because, they are; (i) flexible as they can be applied to different problems with no change in their structure, (ii) simple as they are derived from nature, such as physical phenomena or animal behavior, which rely on straightforward concepts, (iii) easy to be applied as they usually consider problems just like black boxes, (iv) have the ability to search extensively within the search space as well as preventing stagnation in local optima as they have stochastic nature (Sharma and Kaur 2021; Monga et al. 2022). NIO algorithms begin with a random assumptions with no need for calculating the derivative of the search space to obtain the optimal value. Hence, they have become more suitable for real world optimization problems than conventional optimization techniques. Moreover, NIO optimization techniques can discover the optimal solutions to a wide range of problem areas in reasonable time (Sharma and Kaur 2021; Monga et al. 2022; Gao et al. 2020; Sharma and Singh 2020; Singh 2020; Wei et al. 2021; Agrawal et al. 2021; George and Raimond 2013).

Generally, NIO can be broadly classified into; Single-Solution optimization (SSO) and Multi-Solution Optimization (MSO) (Hameed et al. 2021). On SSO, searching for the optimal solution begins with one proposed random candidate solution improved throughout a consequent set of iterations until the optimum solution is achieved. On the other hand, MSO is initialized by a random set of population solutions in a given search space, which are enhanced during the iterations till the best solution is obtained or a stop criteria is achieved. MSO has some advantages over SSO which are; (i) several possible best solutions are available, (ii) MSO has better ability to bypass the local optima problem due to the information sharing among multiple solutions, whereas SSO may trap into local optima which may prevent achieving the global optimum as SSO reforms only one randomly generated solution for a given problem, (iii) MSO can significantly explore the search space with the aid of multiple solutions compares with SSO. NIO algorithms tries to achieve the global optimal solution for a given problem through an adequate balance between two important factors, namely; exploration and exploitation (Sharma and Kaur 2021; Hameed et al. 2021; Mirjalili et al. 2014). Exploration investigates the algorithm ability to discover new search regions for finding global optima, whereas exploitation focuses on finding of optimal solutions (e.g., local optima) within the promising regions (Sharma and Kaur 2021; Mirjalili et al. 2014). Accordingly, extensive exploration does not result in an optimal solution whereas deep exploitation locks up the algorithm in a local optima (Sharma and Kaur 2021; Mirjalili et al. 2014). Due to the stochastic nature of NIOs, it will be difficult to balance between exploration and exploitation. Overexploitation with too little exploration cause the system to converge more quickly, but the true global optimum may not be achieved. Conversely, little exploitation with much exploration cause the search path to wander around in too slow convergence. Hence, a successful NIO algorithm is the one that has the ability to fine-tuning of the two factors to obtain the near-optimal solution.

Despite the rapid development that NIO has achieved recently, not all of the proposed techniques are highly efficient. Only few of them have proven their efficiency and thus achieved widespread fame and popularity in solving real world problems. Moreover, among those popular algorithms, no one performs well in solving all optimization problems. In other words, an algorithm may perform well for some problems while it may perform poorly for others. Based on the source of inspiration, NIO can be classified into three main types, which are; Evolutionary Algorithms (EA) (Gao et al. 2020; Agrawal et al. 2021), Physics and Chemistry based Optimization (PCO) algorithms (Gao et al. 2020; Agrawal et al. 2021), and Swarm Intelligence (SI) algorithms (Monga et al. 2022; Gao et al. 2020; Agrawal et al. 2021). Nature, as an excellent and immense source of inspiration, can help significantly in finding optimal solutions for complex real world problems. In the recent past, the number of SI optimization algorithms in literature has grown considerably (Monga et al. 2022; George and Raimond 2013). SI has become a growing research area that has several open issues, which are not fully addressed. SI deploys the principle of Collective Intelligence (CI) in which a number of agents work cooperatively to accomplish a specific task. To solve an optimization problem using a SI based algorithm, one should scan the huge available algorithms to decide which one is suitable for the problem in hand (Monga et al. 2022; George and Raimond 2013). Some algorithms may introduce superior results in a specific problem, while they may give degraded performance in other problems. The good performance of an algorithm in solving specific problems does not necessarily mean its ability to solve other problems. The reason for this is the different nature of the problems themselves. Hence, as the success of the optimization algorithm depends on the application area, there is a critical need to introduce new SI algorithms that can suit as many as possible optimization problems. Therefore, developing an efficient SI algorithm is an open research issue. This motivates us to develop a novel SI algorithm for efficiently solving wide range of real world optimization problems in a timely manner.

Piranhas are among the most popular fish in the application of cooperative attack and defense strategies. Many voice messages between the herd members allow for cooperation and coordination of work among them, which certainly allows successful hunting and attacking of prey. Piranha is a very popular; however, the controversial behavior of this fish has not been studied in detail. For example, the attack and defense tactics of the piranha fish have not been studied or modeled. The main contribution of this paper is to introduce a new SI optimization algorithm inspired from the attack behavior of Red Piranha fish, hence, it is called Red Piranha Optimization (RPO). The proposed RPO has very limited algorithm parameters and can successfully avoid local optima. Hence, it can be applied in several optimization problems. A case study is also presented by employing the proposed RPO in selecting the most informative features for diagnosing Covid-19. Experimental results have shown that the proposed RPO introduces outstanding performance when compared to its peers of the SI based optimization techniques such as; particle swarm, genetic, gray wolf, ant lion, chimpanzee, whale, fire fly, bat, and sine cosine optimization algorithms. The remainder of the paper is organized as follows; Sect. 2 introduces the proposed RPO technique, and Sect. 3 presents a case study for verifying the high performance and suitability of the proposed RPO. Finally, Sect. 4 concludes the study and outlines the main directions for future work.

## The proposed red piranha optimization (RPO)

Red Piranha Optimization (RPO) is a nature inspired meta-heuristic optimization algorithm which mimics the hunting behavior of Red Piranha fish. Piranha is a schooling, opportunistic ferocious fish (Britannica 2020; Bradford 2017; Mancini 2021). It is omnivorous and has aggressive appetite for meat. Some of their prey includes fish, mollusks, birds, insects, and land animals that enter the water. There are three types of maneuvers associated with feeding piranhas, and they are: (i) Searching, (ii) Encircling, and (iii) Attacking. These maneuvers will be mathematically modeled in the following subsections as the three sequential phases of the proposed RPO algorithm. Although the movement of the piranha swarm is mostly random while searching for prey, this random movement is led by fish with experience in hunting and searching for food called Scouts. When a scouts spot potential prey, it releases a special signal called a "Prey Encircling Signal" (PES) (Britannica 2020; Bradford 2017; Mancini 2021). Hence, the fishes of the flock receive this signal, they begin to surround (Encircle) the prey in the form of spiral movements, the center of which is the prey. When the piranha flock get so close that the prey is within reach of one of the fish, the feeding frenzy begins, as the first fish to reach the prey releases a special signal called "Frenzy Signal" (FS) (Britannica 2020; Bradford 2017; Mancini 2021). Here, the piranha flock regulate its movement, as each fish takes a bite of the prey and makes room for the next fish to take a bite as well in the "Attack-Then-Escape"(ATE) manner until the prey is finished.

### Searching for a prey

While piranha fish search for food, they move in the form of an organized flock in a layered manner. Hence, the weaker and smaller fish are often in the middle to enjoy the greatest protection. On the other hand, the largest and strongest fish in the flock are on the outskirts of the group, where they enjoy protection from the inside, but they represent the outer frame of the group and are usually called group "scouts". Scouts have several tasks which are; (i) guaranteeing the group safety as they represent the outer protective shield, (ii) they are vulnerable to attack in the event of predators presence such as crocodiles, and (iii) scouts represent the group explorers, where they urge the rest of the herd to surround the prey at the moment of discovering a potential one. A scout will broadcast "Encircle Signal" (ES) when a potential prey is detected. When alerted, piranhas are very orderly, hence, they start surrounding the prey to prevent it from moving or escaping. Then, after the prey surrounding has been accomplished the attack begins. Hence, fish follow ATE behavior in which some of the fish will take a bite of the prey and then move aside so another fish can take a bite till the prey becomes a skeleton. With no doubt, successful searching for a prey enhances the exploration ability for the algorithm. In order to accomplish such aim, red piranha schools search randomly according the positions of the school individuals. Since the position of the optimal solution (the prey) is not known, piranha scouts search for prey randomly. A number of scouts are randomly selected to guide the search during the exploration and allow the RPO algorithm to perform a global search. This is quite different from the exploitation phase in which piranhas update their position based on the positions of the leaders (best solutions near to the prey). Hence, the basic key to achieve successful exploration is the randomness. Scouts are the herd leaders in this stage, which are chosen randomly.

Assuming Z to be the total number of iterations, which are uniformly distributed among the three phases of the algorithm (e.g., Searching, Encircling, and Attacking). Hence, the number of iterations for searching, encircling, and attacking, denoted respectively as; ${Z}_{Srch}$, ${Z}_{Enc}$, and ${Z}_{Att}$ can be calculated as; ${Z}_{Srch}=\left\lfloor {\frac{Z}{3}} \right\rfloor$, ${Z}_{Enc}=\left\lfloor {\frac{Z}{3}} \right\rfloor$, while ${Z}_{Att}=\left(Z-2*\left\lfloor {\frac{Z}{3}} \right\rfloor \right)$. For illustration, assuming Z = 10, then ${Z}_{Srch}=\left\lfloor {\frac{10}{3}} \right\rfloor =\left\lfloor {3.33} \right\rfloor \cong 3$, ${Z}_{Enc}=\left\lfloor {\frac{10}{3}} \right\rfloor=\left\lfloor {3.33} \right\rfloor\cong 3$, and ${Z}_{Att}=\left(10-2*3\right)=\left(10-6\right)=4$. Assuming *S* is the set of *n* available solutions (piranha fish), initially, the number of leading scouts (λ) is set, where $\uplambda =\left|\frac{n}{\xi }\right|$ in which 2 ≤ ξ ≤ n is a scaling factor. The next step is to select λ random individuals to be the leading scouts, which are expressed by the set *SCT* = *{sct*_*1*_*, sct*_*2*_*, sct*_*3*_*, sct*_*4*_*,……, sct*_*λ*_*}*. The remaining individuals of the piranha school, which are expressed by the set R = S–L, are randomly categorized into λ clusters. Each cluster has a leading scout as well as $\lceil\frac{n-\lambda }{\lambda }\rceil$ individuals except the last cluster, which may have individuals ≤ $\lceil\frac{n-\lambda }{\lambda }\rceil$. Finally, the position of the *mth* individual that belongs to the *ith* cluster is updated based on the scout of its cluster (e.g., *st*_*i*_). This can be modeled mathematically using (1–4).

1 $${\overrightarrow{D}}_{{P}_{m}}=\left|\overrightarrow{C}.{\overrightarrow{X}}_{{Sct}_{i}}\left(t\right)-{\overrightarrow{X}}_{{P}_{m}}\left(t\right)\right|$$

2 $${\overrightarrow{X}}_{{P}_{m}}\left(t+1\right)={\overrightarrow{X}}_{{Sct}_{i}}\left(t\right)-\overrightarrow{A}.{\overrightarrow{D}}_{{P}_{m}}$$

3 $$\overrightarrow{A}=\overrightarrow{{r}_{1}}*\left(-2+\overrightarrow{{r}_{2}}\right)+\left(1-\overrightarrow{{r}_{1}}\right)\left(1+\overrightarrow{{r}_{3}}\right)$$

4 $$\overrightarrow{C}=2.\overrightarrow{{r}_{4}}$$

where ${\overrightarrow{D}}_{{P}_{m}}$ is the distance between the *m*th piranha fish (*m*th solution) and the prey, ${\overrightarrow{X}}_{{Sct}_{i}}\left(t\right)$ is the position vector of the scout of the *ith* cluster, $\overrightarrow{{r}_{1}}$, $\overrightarrow{{r}_{2}}$, $\overrightarrow{{r}_{3}}$ and $\overrightarrow{{r}_{4}}$ are random vectors in which $\overrightarrow{{r}_{1}}$∈{0,1}, $\overrightarrow{{r}_{2}}$∈[0,1[, ∈$\overrightarrow{{r}_{3}}$]0,1], and $\overrightarrow{{r}_{4}}$∈[0,1], $\overrightarrow{A}$ and $\overrightarrow{C}$ are coefficient vectors. The different steps for the searching phase of the RPO is depicted in algorithm 1. Generally, $\overrightarrow{A}$ has a random value inside the interval [− a,a] in which a decreases from 2 to 0 over the successive iterations. During searching for a prey (e.g., exploration) $\overrightarrow{A}$ has a random values that are greater than 1 or less than − 1. Based on this assumption, piranhas are able to move far away from their randomly chosen reference scout, instead of the best leaders found so far. This allows the fish to perfectly scan the solution domain and discover new regions by moving far away from their reference scout. Hence, RPO algorithm starts from a set of random solutions (random positions of search agents or simply piranha fish). After each iteration, high exploration ability is accomplished due to the position updating mechanism of piranhas using (2). However, during the attacking phase in which similar update position equations will be used, high exploitation and convergence are achieved by allowing the vector $\overrightarrow{A}$ to be in interval [− 1,1]. The behavior of piranhas is simulated by decreasing the value of $\overrightarrow{a}$, which in turn decreases $\overrightarrow{A}$. As $\overrightarrow{A}$ value lies inside the interval [− 1,1], the new position of the piranha will be in somewhere between its current position and the position of its leaders (which roughly represents the place of prey). Accordingly, RPO algorithm insures high local optima avoidance and convergence speed during the iterations as search agents (piranhas) constantly getting close to prey. So, based on the adaptive variation of the search vector $\overrightarrow{A}$ at searching and attacking phases, RPO is considered as a perfect global optimizer. This is because RPO algorithm easily transit between exploration and exploitation. Simply, RPO algorithm can perform a perfect global search during exploration; also it is eliminating local optima during exploitation with minimal internal parameter adjustments. After each iteration of the searching phase, the location vector of each search agent (except the scouts) is updated and the corresponding objective function is calculated, which reflects the agent’s closeness to the potential prey. After finishing ${Z}_{Srch}$ iterations, there exists $\left(\left\lfloor {\frac{Z}{3}} \right\rfloor+1\right)$ different position vectors for each search agent, which are; $\left\lfloor {\frac{Z}{3}} \right\rfloor$ vectors resulted from the search iterations in additional to the initial random position assumed initially for the agent. Piranha is a greedy fish, hence, greedy selection is performed after performing the iterations of the searching phase. To accomplish such aim, the best position of each search agent is accepted. The best position for the search agent *P*_*i*_ is the agent’s position vector that introduces the maximum verification of the given objective function over all other positions of *P*_*i*_ obtained during the searching phase. Then, each agent is allowed to start the encircling phase form its best position. As an illustrative example, assuming we have *n* = *10* search agents (piranha fish) in two dimensional space *x*_*1*_ and *x*_*2*_. It is assumed that *x*_*1*_ and *x*_*2*_ ∈ [− 5,5], which is assumed to be the search domain. By using *ξ* = *4*, the number of scouts; $\uplambda =\lceil\frac{n}{\xi }\rceil=3$, hence, there are three clusters. Assuming the number of iterations (cycles) equals is 11 (Z = 11). Hence, Z_Srch_ = $\left\lfloor {\frac{11}{3}} \right\rfloor=3$, Z_Enc_ = $\left\lfloor {\frac{11}{3}} \right\rfloor=3$, and Z_Att_ = 11–3–3 = 5. The initial position vectors for the 10 search agents are assigned randomly using the formula; $x=l+rand*(u-l)$, where $l$ is the lower value in the considered interval (e.g., − 5), and u is the upper value (e.g., 5). The employed objective function is to minimize $f\left(X\right)={x}_{1}^{2}-{x}_{1}{x}_{2}+{x}_{2}^{2}+2{x}_{1}+4{x}_{2}+3$. Consider Table 1, which summarizes the three iterations during the searching phase.

**Table 1:** Calculations for the illustrative example

Agent	Initially			First iteration						
	Location ($\overrightarrow{X}$)		Objective function value	Is a scout	Scout	Parameters		Location ($\overrightarrow{X}$)		Objective function value
						$\overrightarrow{A}$	$\overrightarrow{C}$	x_1_	x_2_	
	x_1_	x_2_								
P_1_	− 0.451	1.913	14.476	NO	P_2_	− 1.89.45	0.568	4.972	3.582	47.014
P_2_	1.983	0.562	12.348	YES	–	–	–	1.983	0.562	12.348
P_3_	1.446	3.449	28.6878	NO	P_6_	1.766	1.802	− 3.997	− 1.440	1.540
P_4_	3.114	− 3.367	27.279	NO	P_2_	1.987	0.876	− 0.753	− 5.000	3.296
P_5_	1.432	0.488	9.406	NO	P_9_	− 1.235	0.674	5.000	0.185	37.849
P_6_	− 0.345	3.451	29.333	YES	–	–	–	− 0.345	3.451	29.333
P_7_	4.378	− 1.046	32.412	NO	P_2_	1.832	1.566	− 0.3487	− 2.967	− 1.675
P_8_	− 2.876	2.345	27.143	NO	P_6_	− 1.566	0.934	3.654	4.826	48.620
P_9_	4.129	− 2.491	34.833	YES	–	–	–	4.129	− 2.491	34.833
P_10_	4.345	− 3.554	44.426	NO	P_9_	1.331	0.778	2.621	− 4.642	30.258

Agent	Second iteration							Third iteration						
	Is a scout	Scout	Parameters		Location ($\overrightarrow{X}$)		Objective function value	Is a scout	Scout	Parameters		Location ($\overrightarrow{X}$)		Objective function value
			$\overrightarrow{A}$	$\overrightarrow{C}$	x_1_	x_2_				$\overrightarrow{A}$	$\overrightarrow{C}$	x_1_	x_2_	
P_1_	YES	–	–	–	4.972	3.582	47.014	NO	P_8_	1.433	1.561	1.977	− 1.052	9.841
P_2_	NO	P_1_	1.433	1.561	− 3.308	− 3.625	− 6.024	NO	P_8_	1.021	0.464	− 1.89.48	− 1.07	− 2.36
P_3_	NO	P_4_	− 1.421	0.942	3.919	− 0.353	26.293	NO	P_7_	1.366	0.691	4.366	− 0.202	30.909
P_4_	YES	–	–	–	− 0.753	− 5.000	3.296	NO	P_5_	1.278	0.256	1.323	− 3.023	8.442
P_5_	NO	P_10_	− 1.366	1.691	3.397	5.000	49.349	YES	–	–	–	3.397	5.000	49.349
P_6_	NO	P_4_	1.278	1.256	− 1.521	− 5.000	− 0.334	NO	P_5_	1.061	0.872	− 1.36	− 4.931	0.014
P_7_	NO	P1	− 1.061	0.072	5.000	5.000	58	YES	–	–	–	5.000	5.000	58.00
P_8_	NO	P_10_	− 1.211	1.334	2.812	5.000	47.471	YES	–	–	–	2.812	5.000	47.471
P_9_	NO	P_4_	− 1.088	0.131	3.847	− 3.002	34.046	NO	P_7_	1.011	0.934	4.168	− 2.756	36.767
P_10_	YES	–	–	–	2.621	− 4.642	30.258	NO	P_7_	1.188	0.131	2.664	− 1.293	15.369

For illustration, consider the first iteration for the search agent *P*_*1*_ as depicted in Table 1. It can be noticed that Scout (P_1_) is P_2_, ${\overrightarrow{X}}_{{P}_{2}}\left(t\right)=\left(\genfrac{}{}{0pt}{}{+1.983}{+0.562}\right)$, ${\overrightarrow{X}}_{{P}_{1}}\left(t\right)=\left(\genfrac{}{}{0pt}{}{-0.451}{+1.913}\right)$, ${\overrightarrow{C}}_{1}=2*{r}_{4}=2*0.284=0.568$, ${\overrightarrow{A}}_{1}=-\mathrm{1.89.45}$, substitute in (1), ${\overrightarrow{D}}_{{P}_{1}}=\left|{\overrightarrow{C}}_{1}.{\overrightarrow{X}}_{{P}_{2}}\left(t\right)-{\overrightarrow{X}}_{{P}_{1}}\left(t\right)\right|= \left|0.568*\left(\genfrac{}{}{0pt}{}{+1.983}{+0.562}\right)-\left(\genfrac{}{}{0pt}{}{-0.451}{+1.913}\right)\right|=\left(\genfrac{}{}{0pt}{}{1.577}{1.594}\right)$. Then, substitute in (2), ${\overrightarrow{X}}_{{P}_{1}}\left(t+1\right)={\overrightarrow{X}}_{{P}_{2}}\left(t\right)-{\overrightarrow{A}}_{1}.{\overrightarrow{D}}_{{P}_{1}}=\left(\genfrac{}{}{0pt}{}{+1.983}{+0.562}\right)-(-\mathrm{1.89.45})*\left(\genfrac{}{}{0pt}{}{1.577}{1.594}\right)=\left(\genfrac{}{}{0pt}{}{+4.972}{+3.582}\right)$. Finally, the objective function for the new position of the search agent can be calculated, which is 47.014. It is important to mention that when calculating the new position for a search agent (piranha fish), sometimes *x*_*1*_ or *x*_*2*_ is outside the pre-assigned range, which is [− 5,5], for illustration, in the first iteration for the search agent *P*_*4*_, after calculating *x*_*2*_, it is found that *x*_*2*_ = − 7.106, which is outside the legal range (e.g., x_2_ ∉ [− 5,5]).
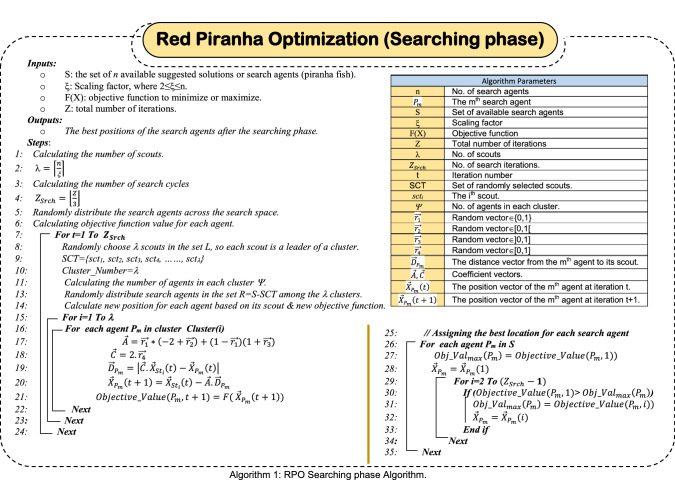


Hence, as seen in Table 1, it is replaced by the minimum value, which is − 5. Also, if the calculated value of *x*_*1*_ or *x*_*2*_ goes above 5 (the maximum legal value), it should be replaced by 5. This situation takes place when calculating *x*_*2*_ for the search agent *P*_*5*_ at the first iteration in which *x*_*2*_ equals 5.797, hence, it is replaced by 5. After finishing the three iterations of the searching phase, there exist four different position vectors for each search agent with the corresponding objective function (three vectors produced through the three iterations of the searching phase in addition to the initial assumed random position). For illustration, consider the four position vectors associated with the search agent *P*_*3*_, which are illustrated in Table 2. On the other hand, the best position vectors for all search agents with the corresponding objective function values are illustrated in Table 3. As was mentioned before, piranha fish are greedy predators, hence, they perform what is meant by greedy selection. Hence, after finishing the searching phase, each search agent (piranha fish) scans back the positions it discovers as well as the corresponding objective function validation level, then it returns to the position that gives the best validation. By depicting Table 3, it can be concluded that the best agent after the searching phase is *P*_*2*_ as it demonstrates the best validation of the considered objective function.

**Table 2:**
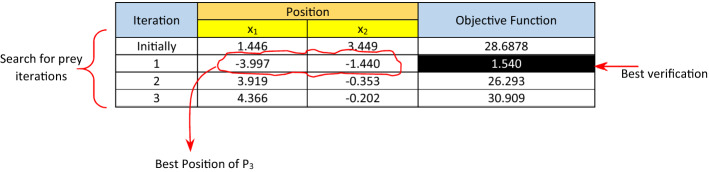
The four position vectors associated with P_3_ in the illustrative example

**Table 3:** The best positions for all agents in illustrative example

Search agent	Position		Objective Function	Rank
	x_1_	x_2_		
P_1_	1.977	− 1.052	9.841	7
P_2_	− 3.308	− 3.625	− 6.024	1
P_3_	− 3.997	− 1.440	1.540	4
P_4_	− 0.753	− 5.000	3.296	5
P_5_	1.432	0.488	9.406	6
P_6_	− 1.521	− 5.000	− 0.334	3
P_7_	− 0.3487	− 2.967	− 1.675	2
P_8_	− 2.876	2.345	27.143	9
P_9_	3.847	− 3.002	34.046	10
P_10_	2.664	− 1.293	15.369	8

### Encircling the prey

During searching for a prey, although the movement of individuals is guided by the scouts, the movement is random in nature (e.g., creative chaos), which allows the agents to discover new areas of the search domain. However, when some fish (called leader or alpha fish as they are the closest to the prey) discover the presence of a potential prey, they issue a certain signal to the rest of the herd members to follow them. This signal is called the "Prey Encircling Signal" (PES). As soon as this signal spreads among the herd individuals, they start surrounding the prey to stop it from moving. Then, after prey encircling has accomplished, the attacking phase begins. Logarithmic spiral has been chosen as the main position update mechanism for herd individuals during the encircling phase. However, any other type of spiral can be employed subject to the following conditions; (i) the initial point of the spiral should start from the search agent (e.g., piranha fish), (ii) the final point of the spiral should be the position of the prey, (iii) spiral fluctuation range should not exceed the search space.
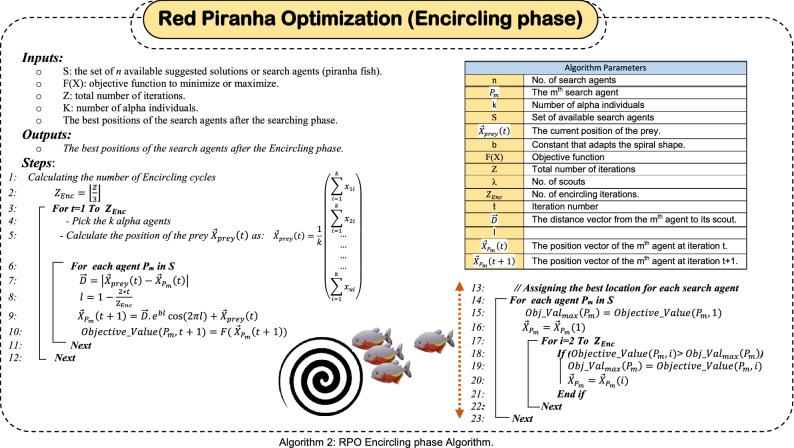


Since the prey represents the optimal solution, and its location is not known, the herd members follow the proposed leaders (alpha fishes), as they are the closest and most aware of the prey’s location. So, the first step is to identify the *k* Alpha individuals, where *k* is hypothetical and its value should not exceed one tenth of the herd size (e.g., 1 ≤ k ≤ $n/10$). It is assumed that the location of the potential prey is in the middle position between the selected *k* alpha fishes, so the hypothetical place of the prey in *u* dimensional space is calculated using (5). Then, for updating the position of each individual in the herd, initially, the distance from the individual to the potential prey is calculated using (6), then, spiral equation is used to describe the movement of the search agents towards the prey as illustrated in (7).

5 $${\overrightarrow{X}}_{prey}\left(t\right)=\frac{1}{k}\left(\begin{array}{c}\sum_{i=1}^{k}{x}_{1i}\\ \sum_{i=1}^{k}{x}_{2i}\\ \dots \\ \dots \\ \dots \\ \dots \\ \sum_{i=1}^{k}{x}_{ui}\end{array}\right)$$

6 $$\overrightarrow{D}=\left|{\overrightarrow{X}}_{prey}\left(t\right)-{\overrightarrow{X}}_{{P}_{m}}\left(t\right)\right|$$

7 $${\overrightarrow{X}}_{{P}_{m}}\left(t+1\right)=\overrightarrow{D}.{e}^{bl}\mathrm{cos}(2\pi l)+{\overrightarrow{X}}_{prey}\left(t\right)$$

8 $$l=1-\frac{2*t}{{\mathrm{Z}}_{\mathrm{Enc}}}$$

where, ${\overrightarrow{X}}_{prey}\left(t\right)$ is the predicted location of the prey at the iteration *t*, ${\overrightarrow{X}}_{{P}_{m}}\left(t\right)$ is the position of the *mth* search agent, $\overrightarrow{\mathrm{D}}$ is the distance between the *mth* search agent and the prey, *b* is a constant defines the shape of the logarithmic spiral, and *l* is a number in the interval [− 1,1] and calculated through the iterations using (8). This also guarantees the good exploitation performed during the encircling phase of the RPO. The sequential steps that should be followed during the encircling phase are illustrated in algorithm 2. Returning again to the illustrative example presented in Sect. 2.1, considering the search agents *P*_*1*_*, P*_*2*_*, …., P*_*10*_ depicted in Table 3. There are three iterations for the encircling phase (e.g., ${\mathrm{Z}}_{\mathrm{Enc}}=3$). Assuming *k* = *3*, hence, the best (alpha) search agents are identified from the herd illustrated in Table 3, which are; *P*_*2*_*, P*_*7*_*, and P*_*6*_ respectively. The location vector for those alpha fishes are; $${\overrightarrow{X}}_{{P}_{2}}\left(t\right)=\left(\begin{array}{c}-3.308\\ -3.625\end{array}\right)$$, $${\overrightarrow{X}}_{{P}_{7}}\left(t\right)=\left(\begin{array}{c}-0.3487\\ -2.967\end{array}\right)$$, and $${\overrightarrow{X}}_{{P}_{6}}\left(t\right)=\left(\begin{array}{c}-1.521\\ -5.000\end{array}\right)$$, hence, the virtual location of the prey is $${\overrightarrow{X}}_{prey}\left(t\right)=\frac{1}{3}\left(\begin{array}{c}-5.178\\ -11.592\end{array}\right)=\left(\begin{array}{c}-1.726\\ -3.864\end{array}\right)$$. Table 4 illustrates the calculations required for the three consecutive iterations of the encircling phase considering the Eqs. (5–8). Results depicted in such table ensures the effectiveness of both the exploration and exploitation performed during the encircling phase as each search agent keeps introducing better objective function validation across the consecutive iterations. This behavior guarantees the effectiveness of RPO algorithm as it moves continuously towards the optimal solution. Again, piranha are greedy fish, hence, at the end of the encircling phase, the search agents move to their best positions. The best position for a search agent is the one that introduces the best validation of the given objective function. By depicting Table 4, the best positions of the 10 search agents at the end of encircling phase are illustrated in Table 5 at which *P*_*6*_ is the closest agent to the prey as it introduces the best objective function validation.

**Table 4:** Different iterations for the encircling phase of the illustrative example

Agent	Initially			First iteration (t = 1) $${\overrightarrow{X}}_{prey}=\left(\begin{array}{c}-1.726\\ -3.864\end{array}\right)$$, l = 0.3333, b = 1				Second iteration (t = 2) $${\overrightarrow{X}}_{prey}=\left(\begin{array}{c}-0.758\\ -2.664\end{array}\right)$$, l = − 0.3333, b = 1				Third iteration (t = 3) $${\overrightarrow{X}}_{prey}=\left(\begin{array}{c}-0.454\\ -2.430\end{array}\right)$$, l = − 1, b = 1			
	Location ($\overrightarrow{X}$)		Objective function value	$\overrightarrow{D}$	Location ($\overrightarrow{X}$)		Objective function value	$\overrightarrow{D}$	Location ($\overrightarrow{X}$)		Objective function value	$\overrightarrow{D}$	Location ($\overrightarrow{X}$)		Objective function value
					x_1_	x_2_			x_1_	x_2_			x_1_	x_2_	
	x_1_	x_2_													
P_1_	1.977	− 1.052	9.841	$$\left(\begin{array}{c}3.703\\ 2.812\end{array}\right)$$	2.482	− 0.669	13.556	$$\left(\begin{array}{c}3.240\\ 1.995\end{array}\right)$$	1.132	− 1.500	4.495	$$\left(\begin{array}{c}1.586\\ 0.931\end{array}\right)$$	− 0.619	− 2.527	− 3.142
P_2_	− 3.308	− 3.625	− 6.024	$$\left(\begin{array}{c}1.582\\ 0.239\end{array}\right)$$	0.072	− 3.592	1.943	$$\left(\begin{array}{c}0.830\\ 0.929\end{array}\right)$$	− 0.274	− 2.122	− 2.039	$$\left(\begin{array}{c}0.180\\ 0.308\end{array}\right)$$	− 0.472	− 2.462	− 2.671
P_3_	− 3.997	− 1.440	1.540	$$\left(\begin{array}{c}2.271\\ 2.424\end{array}\right)$$	0.855	− 1.109	3.182	$$\left(\begin{array}{c}1.613\\ 1.554\end{array}\right)$$	0.183	− 1.757	− 0.220	$$\left(\begin{array}{c}0.637\\ 0.673\end{array}\right)$$	− 0.520	− 2.500	− 2.820
P_4_	− 0.753	− 5.000	3.296	$$\left(\begin{array}{c}0.973\\ 1.136\end{array}\right)$$	− 0.620	− 2.573	− 3.124	$$\left(\begin{array}{c}0.138\\ 0.091\end{array}\right)$$	− 0.678	− 2.617	− 3.292	$$\left(\begin{array}{c}0.224\\ 0.181\end{array}\right)$$	− 0.477	− 2.449	− 2.693
P_5_	1.432	0.488	9.406	$$\left(\begin{array}{c}3.158\\ 4.352\end{array}\right)$$	1.863	1.082	13.676	$$\left(\begin{array}{c}2.621\\ 3.745\end{array}\right)$$	0.771	− 0.479	3.820	$$\left(\begin{array}{c}1.225\\ 1.952\end{array}\right)$$	− 0.582	− 2.634	− 2.955
P_6_	− 1.521	− 5.000	− 0.334	$$\left(\begin{array}{c}0.205\\ 1.136\end{array}\right)$$	− 1.493	− 2.573	− 5.270	$$\left(\begin{array}{c}0.735\\ 0.091\end{array}\right)$$	− 0.329	− 2.611	− 2.037	$$\left(\begin{array}{c}0.124\\ 0.181\end{array}\right)$$	− 0.467	− 2.449	− 2.657
P_7_	− 0.3487	− 2.967	− 1.675	$$\left(\begin{array}{c}1.377\\ \mathrm{0.89.47}\end{array}\right)$$	− 0.161	− 2.845	− 1.040	$$\left(\begin{array}{c}0.597\\ 0.181\end{array}\right)$$	− 0.410	− 2.558	− 2.388	$$\left(\begin{array}{c}0.044\\ 0.128\end{array}\right)$$	− 0.458	− 2.443	− 2.630
P_8_	− 2.876	2.345	27.143	$$\left(\begin{array}{c}1.150\\ 6.209\end{array}\right)$$	− 0.419	3.192	26.629	$$\left(\begin{array}{c}0.339\\ 5.855\end{array}\right)$$	− 0.560	0.753	6.192	$$\left(\begin{array}{c}0.107\\ 3.183\end{array}\right)$$	− 0.465	− 2.762	− 2.416
P_9_	3.847	− 3.002	34.046	$$\left(\begin{array}{c}5.573\\ 0.862\end{array}\right)$$	4.607	− 2.884	43.509	$$\left(\begin{array}{c}5.365\\ 0.221\end{array}\right)$$	2.372	− 2.535	15.670	$$\left(\begin{array}{c}2.826\\ 0.105\end{array}\right)$$	− 0.749	− 2.441	− 3.570
P_10_	2.664	− 1.293	15.369	$$\left(\begin{array}{c}4.390\\ 2.571\end{array}\right)$$	3.263	− 0.942	20.364	$$\left(\begin{array}{c}4.021\\ 1.721\end{array}\right)$$	1.588	− 1.659	7.447	$$\left(\begin{array}{c}2.042\\ 0.771\end{array}\right)$$	− 0.667	− 2.511	− 3.302
${\overrightarrow{X}}_{prey}$	− 1.726	− 3.864		${\overrightarrow{X}}_{prey}$	$-0.758$	$-2.664$		${\overrightarrow{X}}_{prey}$	$-0.454$	$-2.430$		${\overrightarrow{X}}_{prey}$	− 0.678	− 1.870

**Table 5:** Best positions for all agents in illustrative example (after encircling)

Search agent	Position		Objective function	Rank
	x_1_	x_2_		
P_1_	− 0.619	− 2.527	− 3.142	5
P_2_	− 0.472	− 2.462	− 2.671	8
P3	− 0.520	− 2.500	− 2.820	7
P4	− 0.678	− 2.617	− 3.292	4
P5	− 0.582	− 2.634	− 2.955	6
P6	− 1.493	− 2.573	− 5.270	1
P_7_	− 0.458	− 2.443	− 2.630	9
P_8_	− 0.465	− 2.762	− 2.416	10
P_9_	− 0.749	− 2.441	− 3.570	2
P_10_	− 0.667	− 2.511	− 3.302	3

### Attacking the prey

At the end of the encircling phase, the prey has been encircled tightly and is no longer able to escape. Also, the search agents are becoming too close to the prey, and here alpha fishes issue special signal, called Frenzy Signal (FS) to start the attack process. After FS is issued by the leader fish (alpha fishes), the rest of the herd are in a state of frenzy as a result of approaching the prey and compete for reaching it. Hence, they follow the *k* alpha (leader) fish as they are the closest to the prey. Updating the position of each search agent during the attacking phase is accomplished by using (9–13). The *k* Alpha individuals are initially identified, then the predicted location of the potential prey is calculated using (5), which is assumed to be in the middle position of the alpha fishes. Finally, the position of rest of the herd is calculated by initially calculating the distance between the individual and the potential prey using (9), then the new position of the individual is calculated using (10).

9 $${\overrightarrow{D}}_{{P}_{m}}=\left|{\overrightarrow{C}.\overrightarrow{X}}_{prey}\left(t\right)-{\overrightarrow{X}}_{{P}_{m}}\left(t\right)\right|$$

10 $${\overrightarrow{X}}_{{P}_{m}}\left(t+1\right)={\overrightarrow{X}}_{prey}\left(t\right)-\overrightarrow{A}.{\overrightarrow{D}}_{{P}_{m}}$$

11 $$\overrightarrow{A}=2.\overrightarrow{a}.\overrightarrow{{r}_{1}}-\overrightarrow{a}$$

12 $$\overrightarrow{C}=2.\overrightarrow{{r}_{2}}$$

13 $$a=2-t*\frac{2}{{\mathrm{Z}}_{\mathrm{Att}}}$$

where, ${\overrightarrow{X}}_{prey}\left(t\right)$ is the predicted location of the prey at the iteration *t*, ${\overrightarrow{X}}_{{P}_{m}}\left(t\right)$ is the position of the *mth* search agent, ${\overrightarrow{D}}_{{P}_{m}}$ is the distance between the *mth* search agent and the prey, $\overrightarrow{{r}_{1}}$ and $\overrightarrow{{r}_{2}}$ are random vectors ∈ [0,1], $\overrightarrow{a}$ vector decreases linearly from 2 to 0 over the course of iterations, $\overrightarrow{A}$ and $\overrightarrow{C}$ are coefficient vectors. During the attacking phase, $\overrightarrow{A}$ is set to a random value in [− 1,1], hence, the new position of the search agent will be anywhere in between the current position of the search agent and the position of the prey. Now, it is important to discuss a strange behavior of piranha during the attacking phase. As alpha fishes emit the frenzy signal that prompts the rest of the herd to follow because prey is very close. Here the fish of the flock become very close to each other and crowding occurs between them, which we can call "crowding of solutions" and may lead to "solutions collision". As a result of the spread of the frenzy signal among the herd fish, as well as the fish getting too close to each other, the fish become voracious and more aggressive, which may lead the fish to attack each other instead of attacking the prey. This may lead to the loss of some of them as a result of injury or death. And as it is known, each fish represents one of the proposed solutions, and thus, the loss of one of the fish means the loss of one of the potential solutions. Piranhas in nature overcome this problem by escaping from each other if they sense an attack from their peers in the herd, or rather, we can call this wrong attack as; "friendly fire". The escaping fish, which is often weaker than its counterpart, begins to find a safe place and then starts following the prey again by re-tracking the alpha fishes.

In order to model this behavior, we first assume that each fish tries to protect itself by maintaining a safe distance between it and its peers from the herd fish, which is called safety shield. The width of the safety shield is assumed to be *δ*. As a result of this hypothesis, if the distance between two adjacent fish is less than *2δ*, then the two fish are very close to each other. This may allow one of them to attack the other as a result of the spread of the frenzy signal among the fish, which makes the fish very voracious. Of course, when one of the fish (search agent or solution) attacks another, which is called a solutions collision, one of the solutions will be lost. The collision condition between *P*_*G*_ and *P*_*H*_ is *dis(P*_*G*_*,P*_*H*_*)* < *2δ*, where *dis(P*_*G*_*,P*_*H*_*)* is the Cartesian distance between the two search agents *P*_*G*_ and *P*_*H*_ in *u* dimensional space, and *δ* is the width of the safety shield. Assuming ${\overrightarrow{X}}_{{P}_{G}}\left(t\right)=<{x}_{1G}, {x}_{1G}, {x}_{2G}, \dots ..{x}_{uG}>$ and ${\overrightarrow{X}}_{{P}_{H}}\left(t\right)=<{x}_{1H}, {x}_{1H}, {x}_{2H}, \dots ..{x}_{uH}>$, then the Cartesian distance between agents *P*_*G*_ and *P*_*H*_ in *u* dimensional space can be calculated using (14).

14 $$dis\left({P}_{G},{P}_{H}\right)=\sqrt[2]{{\left({x}_{1G}-{x}_{1H}\right)}^{2}+{\left({x}_{2G}-{x}_{2H}\right)}^{2}+\dots \dots . {+ \left({x}_{uG}-{x}_{uH}\right)}^{2}}$$

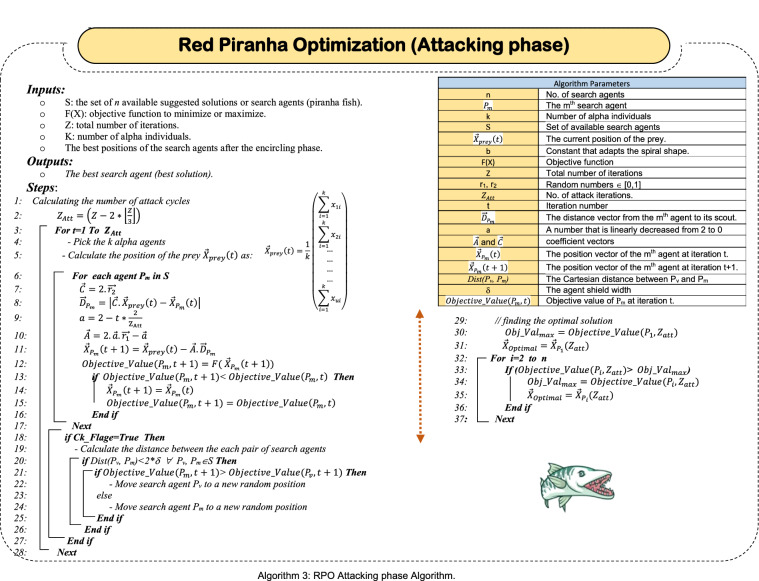


Therefore, after each iteration in the attacking phase, the distance between each pair of the search agents should be calculated to test the collision condition. Hence, when a collision between two (or more) search agents is detected, the most powerful agent (which is the one that maximizes the objective function verification) is kept in its position, while the other agent (weaker one) escapes to another random position to start tracking prey again. However, the frequent detection of collision between search agents after each iteration will definitely affect the speed of execution of the algorithm. To overcome such hurdle, check pointing technique can be used. Hence, collision detection is allowed only at certain iterations not all of them. The number of checkpoints is set a priori, hence, checkpoints can be distributed uniformly, randomly, or follow a specific pattern through the attacking phase iterations. For illustration, checkpoints can be performed after the higher iterations at which the search agents are much closer to the prey and to each other, which allows a higher probability of collisions. On the other hand, if it is needed to distribute the checkpoints uniformly, a suggested technique to perform the ith checkpoint after the ${\left(\left\lfloor {\frac{{Z}_{att}}{{n}_{CK}}} \right\rfloor*i-1\right)}^{th}$ iteration, where ${Z}_{att}$ is the number of iterations of the attacking phase, ${n}_{CK}$ is the number of the required checkpoints. For illustration if ${Z}_{att}=7$ and ${n}_{CK}=3$. Then, the first checkpoint (i.e., i = 1) takes place after the first iteration $\left(\left\lfloor {\frac{7}{3}} \right\rfloor*1-1=1\right)$, the second checkpoint takes place after third iteration $\left(\left\lfloor {\frac{7}{3}} \right\rfloor*2-1=3\right)$, while the third checkpoint takes place at the fifth iteration $\left(\left\lfloor {\frac{7}{3}} \right\rfloor*3-1=5\right)$ and so on. However, as long as the implementation of check pointing may lead to a delay in the execution of the algorithm, its implementation can be optional. Hence, there are two versions of RPO. The first is RPO with check pointing, while the other is RPO without check pointing. The different steps for the attacking phase of the RPO is depicted in algorithm 3.

Returning again to the illustrative example presented in Sect. 2.1, considering the search agents *P*_*1*_*, P*_*2*_*, …., P*_*10*_ depicted in Table 5. There are four iterations for the attacking phase (e.g., ${\mathrm{Z}}_{\mathrm{Att}}=4$). For simplicity, no checkpointing is allowed during the attacking phase. Assuming *k* = *3*, hence, the best (alpha) search agents are identified from the herd illustrated in Table 6, which are; *P*_*6*_*, P*_*9*_*,* and* P*_*10*_. The location vector for those alpha fishes are; $${\overrightarrow{X}}_{{P}_{6}}\left(t\right)=\left(\begin{array}{c}-1.493\\ -2.573\end{array}\right)$$, $${\overrightarrow{X}}_{{P}_{9}}\left(t\right)=\left(\begin{array}{c}-0.749\\ -2.441\end{array}\right)$$, and $${\overrightarrow{X}}_{{P}_{10}}\left(t\right)=\left(\begin{array}{c}-0.667\\ -2.511\end{array}\right)$$, hence, the virtual location of the prey is $${\overrightarrow{X}}_{prey}\left(t\right)=\frac{1}{3}\left(\begin{array}{c}-2.909\\ -7.525\end{array}\right)=\left(\begin{array}{c}-0.970\\ -2.508\end{array}\right)$$. Table 6 illustrates the calculations required for the four consecutive iterations during the attacking phase considering the Eqs. (9–13). Results depicted in Table 6 ensures the effectiveness of the exploitation performed during the attacking phase as each search agent keeps introducing better objective function validation across the consecutive iterations. This behavior guarantees the effectiveness of RPO algorithm as it moves continuously towards the optimal solution.

**Table 6:** Different iterations for the attacking phase of the illustrative example

Agent	Initially			First iteration (t = 1)$${\overrightarrow{X}}_{prey}=\left(\begin{array}{c}-0.970\\ -2.508\end{array}\right)$$					Second iteration (t = 2) $${\overrightarrow{X}}_{prey}=\left(\begin{array}{c}-1.513\\ -2.991\end{array}\right)$$				
	Location ($\overrightarrow{{X}_{{p}_{m}}}$)		Objective function value	$\overrightarrow{A}$	$\overrightarrow{C}$	Location ($\overrightarrow{{X}_{{p}_{m}}}$)		Objective function value	$\overrightarrow{A}$	$\overrightarrow{C}$	Location ($\overrightarrow{{X}_{{p}_{m}}}$)		Objective function value
	x_1_	x_2_				x_1_	x_2_				x_1_	x_2_	
P_1_	− 0.619	− 2.527	− 3.142	0.237	1.689.4	− 1.211	− 2.914	− 4.650	0.982	0.877	− 1.627	− 3.276	− 5.308
P_2_	− 0.472	− 2.462	− 2.671	0.582	1.411	− 1.491	− 3.136	− 5.145	0.0933	0.0842	− 1.640	− 3.260	− 5.350
P_3_	− 0.520	− 2.500	− 2.820	− 0.459	1.872	− 0.375	− 1.501	− 1.923	0.0441	0.763	− 1.547	− 3.026	− 5.330
						− 0.520	− 2.500	− 2.820					
P_4_	− 0.678	− 2.617	− 3.292	0.099	0.084	− 1.029	− 2.747	− 4.267	0.921	0.89.41	− 1.807	− 3.066	− 5.753
P_5_	− 0.582	− 2.634	− 2.955	0.671	1.5	− 1.555	− 3.266	− 5.169	0.341	1.2	− 1.602	− 3.102	− 5.393
P_6_	− 1.493	− 2.573	− 5.270	0.578	1.903	− 1.173	− 3.780	− 3.236	0.982	1.562	− 2.682	− 3.867	− 6.056
						− 1.493	− 2.573	− 5.270					
P_7_	− 0.458	− 2.443	− 2.630	0.296	1.533	− 1.274	− 2.923	− 4.797	− 0.551	0.0931	− 0.889.4	− 1.534	− 3.134
											− 1.274	− 2.923	− 4.797
P_8_	− 0.465	− 2.762	− 2.416	0.432	1.344	− 1.332	− 2.772	− 4.986	0.762	1.236	− 1.923	− 3.697	− 5.379
P_9_	− 0.749	− 2.441	− 3.570	0.821	0.638	− 1.077	− 3.199	− 4.001	0.782	0.911	− 1.749	− 3.362	− 5.465
P_10_	− 0.667	− 2.511	− 3.302	0.761	1.298	− 1.420	− 3.075	− 5.034	− 0.412	1.114	− 1.404	− 2.885	− 5.103
${\overrightarrow{X}}_{prey}$	− 0.970	− 2.508		${\overrightarrow{X}}_{prey}$		− 1.513	− 2.991		${\overrightarrow{X}}_{prey}$		− 2.079	− 3.432	

Agent	Third iteration (t = 3)$${\overrightarrow{X}}_{prey}=\left(\begin{array}{c}-2.079\\ -3.432\end{array}\right)$$					Fourth iteration (t = 4) $${\overrightarrow{X}}_{prey}=\left(\begin{array}{c}-2.041\\ -3.106\end{array}\right)$$				
	$\overrightarrow{A}$	$\overrightarrow{C}$	Location ($\overrightarrow{{X}_{{p}_{m}}}$)		Objective function value	$\overrightarrow{A}$	$\overrightarrow{C}$	Location ($\overrightarrow{{X}_{{p}_{m}}}$)		Objective function value
			x_1_	x_2_				x_1_	x_2_	
P_1_	0.093	1.091	− 2.139	− 3.475	− 5.960	0.094	0.105	− 2.222	− 3.402	− 6.100
P_2_	0.060	0.235	− 2.148	− 3.579	− 5.877	0.872	1.234	− 2.364	− 3.327	− 6.243
P_3_	− 0.099	0.0851	− 1.944	− 3.162	− 5.906	0.295	1.701	− 2.492	− 3.732	− 6.074
P_4_	0.086	0.209	− 2.197	− 3.634	− 5.882	0.796	1.342	− 2.472	− 3.532	− 6.218
P_5_	− 0.512	0.711	− 2.016	− 3.093	− 6.009	0.0911	0.013	− 2.222	− 3.384	− 6.111
P_6_	0.098	0.404	− 2.260	− 3.675	− 5.912	− 0.091	1.108	− 2.041	− 3.085	− 6.035
			− 2.682	− 3.867	− 6.056					
P_7_	− 0.511	0.652	− 2.038	− 3.081	− 6.033	0.392	1.233	− 2.229	− 3.399	− 6.108
P_8_	0.099	1.568	− 2.212	− 3.598	− 5.936	0.116	1.98	− 2.253	− 3.402	− 6.129
P_9_	− 0.567	0.832	− 2.069	− 3.144	− 6.053	0.951	0.882	− 2.296	− 3.491	− 6.113
P_10_	0.018	0.554	− 2.084	− 3.449	− 5.913	0.076	0.087	− 2.186	− 3.348	− 6.095
	${\overrightarrow{X}}_{prey}$		− 2.041	− 3.106		End of Iteration, Optimal Solution is $$\left(\begin{array}{c}-2.364\\ -3.327\end{array}\right)$$				

Piranha are greedy fish, hence, after each iteration, each search agent compares its new position with the old position, and then it chooses the better. This behavior is different from that during the search and encircling phase at which the search agent chooses the best position at the end of the phase. The cause is that the searching phase is dedicated totally for exploration. Hence, the agent is allowed to continue randomly to discover new regions. Although encircling phase is employed for exploitation, the search agent is also allowed to move even if the new position is worse than the old one to give also some support for exploration. Then, the agent chooses the best position at the end of the encircling phase. On the other hand, the attacking phase is dedicated for exploitation, hence, when a search agent exists in a better position than the new one, it reserves the old position preparing to attack the prey. Comparing positions is done based on the objective function validation. Hence, the more the objective function validation, the more the position priority. By depicting Table 6, it can be concluded that each search agent reserves its maximum validation value of the objective function as well as the corresponding position across the successive iterations of the attacking phase. Hence, the best validation value of the objective function for all agents will be at ${Z}_{att}^{th}$ (e.g., final) iteration. As depicted in Table 6, P2 introduces the best position as it introduces the best objective function validation at the final iteration of the attacking phase. Hence, *P*_*2*_ is the optimal solution.

Thus, the efficiency of RPO algorithm has been proven in the continuous case, as after five iterations, the RPO algorithm reached a good value where convergence occurs. Additionally, to prove the efficiency of the RPO algorithm in the binary search space, it will be presented in the next subsection called a case study. To examine the efficiency of RPO in a binary search space during the next subsection, the considered problem is selecting the best set of features for Covid-19 diagnosis.

### Case study: feature selection using binary red piranha optimization (BRPO)

Feature selection is the process of reducing the number of input variables by selecting a subset of consistent, non-redundant, and relevant features when developing a predictive model. Hence, feature selection can reduce the computational cost of the model as well as improving the performance of classification model. In fact, selecting the best subset of features before learning the classification model can improve its performance where it enables the classifier to perform its tasks well and fast. Thus, many of general frameworks consists of two main phases, namely; (i) Feature Selection Phase (FSP) and (ii) Classification Phase (CP). The effectiveness of the proposed RPO will be tested in FSP through selecting the most important features for diagnosing Covid-19 patients. The input of FSP is the training dataset in the form of a set of labeled laboratory tests for people infected with the Covid-19 and other tests of healthy individuals. During FSP, the most informative features for Covid-19 diagnose will be selected using the proposed RPO. Then, in CP, the classical Naïve Bayes (NB) classifier will be trained using the dataset with the most important features for the diagnostic process (Rabie et al. 2019; Saleh and Rabie 2023a). For diagnosing a new case, the selected features will be extracted from the laboratory test of that undiagnosed case. Then, the case will be diagnosed (classified) either to "Healthy" class or to "Infected" class. It is important to mention that the used version of RPO in the feature selection problem can be called Binary Red Piranha Optimization (BRPO). In BRPO, each fish (solution) is represented by a binary vector *X* = *(x*_*1*_*, x*_*2*_*,……, x*_*f*_*)*, *x*_*i*_ ∈ [0,1] in *f* dimensional space. Each dimension represents a feature. Hence, "0" value in the *ith* dimension indicates that the corresponding *ith* feature is not included in the selected set, while "1" value indicates that the *ith* feature is included in the solution. Thus, there are many sequential steps to implement the proposed BRPO to select the best subset of feature from the Covid-19 dataset. BRPO begins with a Swarm (*S*) which contains a set of search agents (or piranhas) denoted by *X*, for example, *S* contains *n* of search agents, therefore,* S* contains *X* = *{X*_*1*_*,X*_*2*_*,….,X*_*m*_*,….,X*_*n*_*}*. Each piranha fish (*X*_*m*_) in *S* represents a potential solution (i.e. a subset of the most efficient features in the dataset) in the *f*-dimensional search agent where *f* represents the total number of features in the Covid-19 dataset, thus, the *mth* piranha fish can be represented as; *X*_*m*_ = *(X*^*1*^_*m*_*, X*^*2*^_*m*_*,….., X*^*f*^_*m*_*)*. The position (bit) value of each piranha fish (*X*_*m*_) is a binary value that may equal one or zero where one indicates the *ith* feature is selected while zero indicates the *ith* feature is deleted; *i* = *{1,2,…,f}*. After an *S* is randomly generated with *n* of search agents in a binary space, the evaluation process should be performed on these search agents using the NB classifier accuracy value as standard classification model to be a fitness function. The mathematical model of the fitness (evaluation) function can be represented using (15).

15 $$Fit \left({X}_{m}\right)=NB\_Accuracy\left({X}_{m}\right)$$

where *Fit(X*_*m*_*)* is the fitness value of the *mth* search agent and *NB_Accuracy(X*_*m*_*)* is the NB classifier accuracy based on the subset of selected features in the *mth* search agent. According to the evaluation values, the best solution is the one that can achieve the maximum accuracy value. In the other words, maximizing the fitness value (classifier accuracy) is the main objective of the feature selection process. After the initial swarm is generated using *n* of search agents in a binary space and these search agents are then evaluated using the accuracy of the NB classifier, the scaling factor (*ξ*) and the number of iterations (*Z*) should be assigned. Based on the value of *ξ*, the number of scouts or clusters ($\lambda$) will be calculated using $\lambda =\lceil\frac{n}{\xi }\rceil$. Then, the remaining search agents in *S* will be randomly distributed among $\lambda$ clusters taking the scout of each cluster as a leader to the remaining search agents within that cluster. Based on the value of *Z*, the number of iterations per the searching, encircling, and attacking phases should be calculated using ${Z}_{Srch}=\left\lfloor {\frac{Z}{3}} \right\rfloor$, ${Z}_{Enc}=\left\lfloor {\frac{Z}{3}} \right\rfloor$, and ${Z}_{Att}=\left(Z-2*\left\lfloor {\frac{Z}{3}} \right\rfloor \right)$ respectively. According to ${Z}_{Srch}$, ${Z}_{Enc}$, and ${Z}_{Att}$, the steps to implement the proposed BRPO are organized in a three parts, namely; (i) steps of the searching phase, (ii) steps of the encircling phase, and (iii) steps of the attacking phase. Initially, steps of the searching phase will be executed until the number of iterations (${Z}_{Srch}$) is terminated. If the ${Z}_{Srch}$ is not finished, then the position of the *mth* individual belonging to the *cth* cluster will be updated based on the scout of its cluster (e.g., *st*_*c*_) using (2). The new position of the *mth* individual; *X*_*m*_ = *(X*^*1*^_*m*_*, X*^*2*^_*m*_*,…..,X*^*f*^_*m*_) includes continuous values, and therefore, a sigmoid function must be used to convert this position into binary values using (16) (Saleh and Rabie 2023b).

16 $${X}_{binary\_m}^{i}\left(t+1\right)= \left\{\begin{array}{c}1 if rand(\mathrm{0,1})\ge sigmoid({X}_{m}^{i})\\ \\ 0 otherwise\end{array}\right.$$

where *X*^*i*^_*binary_m*_* (t* + *1)* is the binary value of *mth* individual at *i*th bit in the next iteration *t* + *1*; *i* = *1,2,3,…,f* and *rand(0,1)* represents a random value between [0,1]. Additionally, *sigmoid(X*^*i*^_*m*_*)* is the sigmoid function that represents the probability of *ith* bit in which it takes 0 or 1 value calculated by using (17) Saleh and Rabie 2023b).

17 $$sigmoid\left({X}_{m}^{i}\right)=\frac{1}{1+{e}^{-{X}_{m}^{i}}}$$

where the base of the natural logarithm is *e*. Each individual in *S* is evaluated using the fitness function in (15) based on the new position *X*^*i*^_*binary_m*_* (t* + *1)* for each individual. In fact, each individual in *S* will store updated position and objective (fitness) values during their journey in search of prey. Steps of the searching phase will be continued until the ${Z}_{Srch}$ is completed. At the end of the searching phase for a prey, the best position of each individual in *S* will be the one that enables the individual to give the best fitness value during their journey in search of prey. In the second part, the steps of the encircling phase will be performed on the best positions of the search agents in *S* given from the searching phase until the number of iterations (${Z}_{Enc}$) is terminated. The first step in the encircling phase is to set number of leaders or alpha fishes (*k*). Then, the best *k* of the search agents will be determined based on their fitness values to use their positions to calculate the position of the potential prey ${\overrightarrow{X}}_{prey}\left(t\right)$ using (5). The position of the prey; *X*_*prey*_ = *(X*^*1*^_*prey*_*, X*^*2*^_*prey*_*,….,X*^*f*^_*prey*_) contains continuous values, and therefore, a sigmoid function must be used to convert this position into binary values using (16). In the next step, the search agents in the swarm will update their positions according to the position of the prey. If the ${Z}_{Enc}$ is not finished, then the position of the *mth* search agent will be updated based on the position of the prey using (7). New position of the *mth* search agent will be converted from continuous to binary values using (16). Then, the new positions of search agents in the swarm will be evaluated using the fitness function in (15). Before starting the next iteration, new *k* of alpha fishes will be determined based on the best fitness values to calculate the current position of the prey using (5). Steps of the encircling phase will be continued until the ${Z}_{Enc}$ is completed.

In the third and final part, the steps of the attacking phase will be performed on the best positions of the search agents given from the encircling phase until the number of iterations (${Z}_{Att}$) is terminated. As in the first step of the encircling phase, the attacking phase begins with setting number of leaders or alpha fishes (*k*). Then, the best *k* of the search agents will be determined based on the best fitness values to use their positions to calculate the position of the potential prey ${\overrightarrow{X}}_{prey}\left(t\right)$ using (5). The position of the prey; *X*_*prey*_ = *(X*^*1*^_*prey*_*, X*^*2*^_*prey*_*,….,X*^*f*^_*prey*_) contains continuous values, and therefore, a sigmoid function must be used to convert this position into binary values using (16). Next, the search agents in the swarm will update their positions according to the position of the prey. If the ${Z}_{Att}$ is not finished, then the position of the *mth* search agent will be updated based on the position of the prey using (10). New position of the *mth* search agent contains continuous values, thus, these values will be converted to binary values using (16). Then, the fitness function in (15) will be used to evaluate the new positions of search agents. Before starting the next iteration, new *k* of alpha fishes will be determined based on the best fitness values to calculate the current position of the prey using (5). Additionally, it is assumed that the collision condition does not satisfied. Steps of the attacking phase will be continued until the ${\mathrm{Z}}_{\mathrm{Att}}$ is completed. Finally, the fittest piranha fish represents the best solution that includes the best subset of features. It is noted that dividing the iterations on the three phases (searching, encircling, and attacking) enables the BRPO algorithm to provide fast and accurate subset of features. The reason is that this process gives an opportunity for each phase to try several times to reach the best solution before implementing the next phase attempting to try to approach the prey, which helps the following phases to be implemented more quickly and accurately.

#### The used dataset and employed parameters

In this subsection, the dataset used to implement the proposed RPO algorithm compared to the most popular optimization algorithms will be described. Then, the parameters employed and their values during execution will be introduced. In fact, the used dataset for this study is called Albert Einstein dataset because it was collected from Albert Einstein Hospital in Brazil and made publicly available by Kaggle (Kaggle 2021). From March 28, 2020 to April 3, 2020, the Albert Einstein dataset consisting of 5644 cases was collected. This informative dataset includes several clinical tests such as urine, rt-PCR, blood, and SARS-CoV-2 test as it contains 110 features (attributes).

The features are Patient ID, Patient age quantile, SARS-Cov-2 exam result, Patient admitted to regular ward (1 = yes, 0 = no),Patient admitted to semi-intensive unit (1 = yes, 0 = no),Patient admitted to intensive care unit (1 = yes, 0 = no), Hematocrit, Hemoglobin, Platelets, Mean platelet volume,Red blood Cells,Lymphocytes,Mean corpuscular hemoglobin concentration (MCHC),Leukocytes, Basophils, Mean corpuscular hemoglobin (MCH),Eosinophils, Mean corpuscular volume (MCV) Monocytes, Red blood cell distribution width (RDW),Serum Glucose Respiratory Syncytial Virus,Influenza A,Influenza B, Parainfluenza 1,CoronavirusNL63, Rhinovirus/Enterovirus, Mycoplasma pneumoniae, Coronavirus HKU1,Parainfluenza 3,Chlamydophila pneumoniae,Adenovirus,Parainfluenza 4,Coronavirus229E,CoronavirusOC43,Inf A H1N1 2009,Bordetella pertussis, Metapneumovirus, Parainfluenza 2,Neutrophils,Urea,Proteina C reativa mg/dL,Creatinine, Potassium, Sodium,Influenza B rapid tastefulness A, rapid test, Alanine transaminase, Aspartate transaminase,Gamma-glutamyltransferase,Total Bilirubin, Direct Bilirubin, Indirect Bilirubin, Alkaline phosphatase, Ionized calcium,Strepto A, Magnesium, pCO2 (venous blood gas analysis),Hb saturation (venous blood gas analysis),Base excess (venous blood gas analysis),pO2 (venous blood gas analysis),Fio2 (venous blood gas analysis),Total CO2 (venous blood gas analysis),pH (venous blood gas analysis),HCO3 (venous blood gas analysis),Rods #,Segmented,Promyelocytes, Metamyelocytes, Myelocytes, Myeloblasts, Urine—Esterase, Urine—Aspect, Urine—pH, Urine—Hemoglobin, Urine—Bile pigments, Urine—Ketone Bodies, Urine—Nitrite, Urine—Density, Urine—Urobilinogen, Urine—Protein, Urine—Sugar, Urine—Leukocytes, Urine—Crystals, Urine—Red blood cells, Urine—Hyaline cylinders, Urine—Granular cylinders, Urine—Yeasts, Urine—Color, Partial thromboplastin time (PTT),Relationship (Patient/Normal), International normalized ratio (INR),Lactic Dehydrogenase, Prothrombin time (PT), Activity VitaminB12,Creatine phosphokinase (CPK),Ferritin, Arterial Lactic Acid, Lipase dosage-Dimer, Albumin saturation (arterial blood gases),pCO2 (arterial blood gas analysis),Base excess (arterial blood gas analysis),pH (arterial blood gas analysis),Total CO2 (arterial blood gas analysis), HCO3 (arterial blood gas analysis),pO2 (arterial blood gas analysis),Arterial Fio2, Phosphor, and ctO2 (arterial blood gas analysis).

This dataset includes two main class categories in the SARS-CoV-2 attribute called “positive” and “negative”. While positive class category refers to people infected with Covid-19, negative class refers to non-infected people with Covid-19. In fact, the Albert Einstein dataset includes 559 positive cases and 5085 negative cases (Kaggle 2021). Actually, the proposed BRPO algorithm will be implemented on the Albert Einstein dataset to select the features that most influence the diagnosis of Covid-19. The performance of BRPO will be evaluated against nine of recent binary optimization techniques to select the best subset of features. These optimization techniques are Binary Particle Swarm Optimization (BPSO) (Sharma and Kaur 2021; Harrison et al. 2017), Binary Genetic Algorithm (BGA) (Saleh and Rabie 2023a; Saleh et al. 2016), Binary Gray Wolf Optimization (BGWO) (Sharma and Kaur 2021; Mirjalili et al. 2014), MUDE (Sharma and Kaur 2021; Tan et al.^2022^), and Binary Chimpanzee Algorithm (BCA) (Khishe and Mosavi 2020). Additionally, the rest of the nine optimization techniques are Cat and Mouse-Based Optimizer (CMBO) (Dehghani et al. 2021), Tuna swarm optimization (TSO) (Xie et al. 2021), Pelican Optimization Algorithm (POA) (Trojovský and Dehghani 2022), and White Shark Optimizer (WSO) (Braik et al. 2022). The values of the employed parameters for these ten optimization techniques should be assigned during execution as presented in Table 7. There are many common parameters that have the same values for all optimization techniques.

**Table 7:** The employed parameters and their values during execution

Algorithm	Parameter	Value
BRPO	Number of Scouts ($\uplambda$) (during searching phase)	10,15,20
	Number of alpha fishes (k) (leader fishes during encircling and attacking phases)	5,7,10
	A number defines the shape of the movement logarithmic spiral during the encircling phase (b)	1,2,3
	Number of checkpoints (n_ck_) in the case if there is a collision	3,7,9
BPSO (Sharma and Kaur 2021; Harrison et al. 2017)	Inertia Weight (w)	0.5
	Personal Learning Coefficient (C_1_)	2
	Global Learning Coefficient (C_2_)	2
BGA (Saleh and Rabie 2023a; Saleh et al. 2016),	Probability of selection (P_s_) (using Roulette Wheel)	Random [0,1]
	Probability of crossover (P_c_) (using one point crossover)	Random [0,1]
	Probability of mutation (P_m_) (using one bit flip)	Random [0,1]
BGWO (Sharma and Kaur 2021; Mirjalili et al. 2014)	The encircling coefficient (a)	From 2 to 0
	Random Vectors; r_1_ and r_2_	Random [0,1]
MUDE(Tan et al.^2022^)	Dimension	D = 30
	Population size: NP	NP = 100
	Crossover: CRi	CRi = N(uCRi 0.1);
BCA (Khishe and Mosavi 2020)	The encircling coefficient (f)	From 2 to 0
	Random Vectors; r_1_ and r_2_	Random [0,1]
CMBO (Dehghani et al. 2021)	r is a random value	Random between [0,1]
TSO (Xie et al. 2021)	NP: is the number of tuna populations	50
	α1 and α2 are the coefficients of weight that used to control the tendency of individuals to move towards the optimal individual and the previous individual	α1 = a + (1 − a) • t /tmax α2 = (1 − a) − (1 − a) • t/ tmax
	a is a constant used to measure the extent to which the tuna follow the optimal individual and the previous individual in the initial phase	0.7
	b is a random number	Random value between [0,1]
	TF is a random number	1 or − 1
	Z:each individual randomly chooses one of the two foraging strategies to execute, or chooses to regenerate the position in the search space according to probability z	0.05
PSO (Trojovský and Dehghani 2022)	m is the number of problem variables	Random between [0,1]
	rand is a random number	Random between [0,1]
	s a random number	1 OR 2
	R is a constant	0.2
	Cognitive and social constant (C1, C2)	(2, 2)
WSO (Braik et al. 2022)	a: the acceleration factor which is constant From 2 to 0	0.0005
	r is a random value	Random [0,1]
	c1 and c2 are two uniformly created random numbers	Random [0,1]
	τ denotes the coefficient of acceleration	4.125
	r1, r2 and r3 are random values	in the range of [0, 1]

The parameters were chosen for each algorithm, which provides the highest accuracy according to the conditions set by this author in his research. Also, there was unification of the work environment through the use of the same dataset and the same characteristics, and the implementation of all algorithms on the same device with the same capabilities. Therefore, we compare our proposed method with other methods in the best accuracy for them. Thus, Table 8 is provided to include the common parameters and their values. As presented in Table 8, the common parameters for all techniques are (i) the total number of iterations (z) that is set to 50, 100, 150, 200, 250, 300, 350, 400, 450, and 500, (ii) the number of search agents or individuals (n) that is set to 25, 50, 75, and 100, and (iii) the dimensions of each search agent (f) that equals 110. Except for BGA, the random value (rand) in the sigmoid function belonging to (0,1) used to convert the continuous positions to binary for all used optimization techniques is presented in Table 8 as a common parameter. According to these values of parameters, the performance of the used optimization techniques against the proposed BRPO should be measured to determine the best technique as a feature selection method. Hence, the evaluation metrics will be discussed in the next subsection.

**Table 8:** The common parameters and their values during execution

Parameter	Description	Assigned value
z	Number of iterations	50,100, 150, 200, 250, 300, 350, 400, 450, 500
n	Number of search agents	25, 50, 75, 100
f	Number of features for Covid-19 diagnosis (dimensions)	110
v	Number of accepted features (after optimization)	Calculated based on the applied optimization technique
rand	A random value in the sigmoid function	Random [0,1]

#### Evaluation metrics

In this subsection, accuracy (fitness value), execution time, micro average precision, micro average recall, macro average precision, macro average recall, and f-measure are used as evaluation metrics for all optimization techniques. The main objective of these metrics is to determine the best optimization techniques that can quickly select the best subset of features from the Albert Einstein dataset. In fact, the best optimization technique is the one that provides the maximum accuracy value and the minimum execution time compared to other techniques. The Albert Einstein dataset should be divided into two main subsets of data called training and testing. To calculate the accuracy of each optimization technique, NB classifier as a standard classification method should be learned by using the training set of data based on the selected features from each optimization technique (Rabie et al. 2019; Saleh and Rabie 2023a). Then, NB should tested by using the testing set of data based on the selected features. Finally, confusion matrix is used to calculate the accuracy of NB according to the selected features from each optimization technique (Saleh and Rabie 2023a, 2023b). Accuracy is the number of times cases (testing cases) are correctly classified relative to the total number of cases (testing cases). Thus, the accuracy of *qth* optimization algorithm based on NB classifier can be calculated using (18).

18 $$Accuracy\left({opt\_algorithm}_{q}\right)=Max (NB\_accuracy({X}_{m}))$$

where $Accuracy\left({opt\_algorithm}_{q}\right)$ is the accuracy of *qth* optimization algorithm; q = 1,2,….,10. $Max (NB\_accuracy({X}_{m}))$ is the maximum NB accuracy value provided by the best search agent *X*_*m*_ where *m* = *1,2,…,n* and *n* is the total number of search agents. The accuracy of *mth* search agent in the population or swarm can be calculated using (19).

19 $$NB\_accuracy({X}_{m})= \frac{the \;number\; of \;correct \;classifications}{total \;number \;of \;classifications }*100$$

The second evaluation metric is the execution time that represents the multiplication of the number search agents by the number of iterations using (20).

20 $$Execution time\left({opt\_algorithm}_{q}\right)= n*z$$

where $Execution time \left({opt\_algorithm}_{q}\right)$ is the execution time of the *qth* optimization algorithm, *n* is the number of search agents and *z* is the number of iterations that depends on the stop condition ($Accuracy\left({opt\_algorithm}_{q}(t)\right)-Accuracy\left({opt\_algorithm}_{q}(t+1)\right)<{10}^{-7})$. Where *t* is the current iteration number and *t* + *1* is the next iteration number. Thus, *z* may contain the number of iterations (*z*_*o*_) which is less than the total iterations number; z = *z*_*o*_ if the stop condition is matched, otherwise, it is equal to the total number of iterations. In the next subsection, the experimental results will be evaluated using both evaluation metrics called accuracy and execution time.

### Experimental results

In this subsection, the experimental results for measuring the superiority of BRPO compared to the other nine algorithms given in Table 7 will be provided to quickly and accurately select the best features. In fact, the experimental results will be generated through two scenarios, which are; (i) testing the performance of BRPO according to the number of iterations, the number of search agents, and the collision detection. And (ii) providing a comparative study to test the best results of BRPO against the other nine algorithms given in Table 7. In the first scenario, the accuracy, and execution time of BRPO will be measured by using (19) and (20) respectively. These measurements will be calculated according to many numbers of iterations (z) and many numbers of search agents (n) presented in Table 8. Depending on the collision detection, BRPO will be executed based on two states called “without collision” and “with collision”. In the without collision state, the performance of BRPO (accuracy and execution time) will be measured without taking the collision between fishes in the calculation and also without taking into account the number of checkpoints.

On the other hand, in the with collision state, the performance of BRPO (accuracy and execution time) will be measured by taking the collision between fishes in the calculation and also by taking into account the number of checkpoints (n_ck_) presented in Table 8. In the second scenario, a comparative study between BRPO and the other nine algorithms given in Table 7 will be introduced by calculating the accuracy, execution time, micro average precision,micro average recall, macro average precision, macro average recall, and f-measure of them. These calculations will be performed according to many numbers of iterations (z) at each specified number of search agents separately. In fact, the ten optimization algorithms are executed as wrapper feature selection methods to select the best features that enable the NB classifier to quickly and correctly classify Covid-19 patients. Our implementation uses Albert Einstein dataset to select the best features from 110 features which have a significant impact on Covid-19 patients (Kaggle 2021).

- **Testing the performance of Binary Red Piranha Optimization (BRPO) Algorithm**

**Fig. 1:**
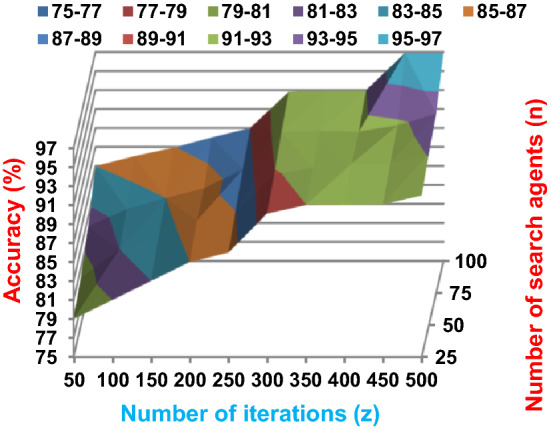
The accuracy of BRPO without collision

**Fig. 2:**
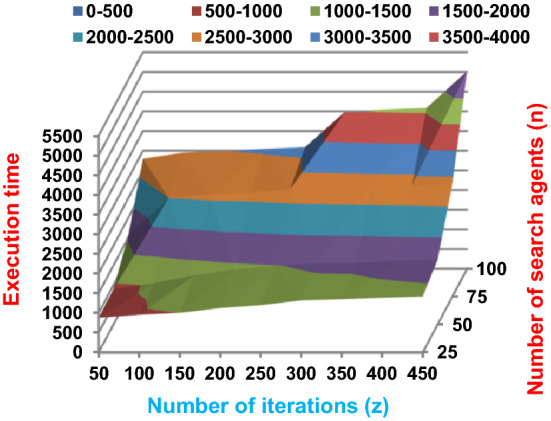
The speed of BRPO without collision

**Fig. 3:**
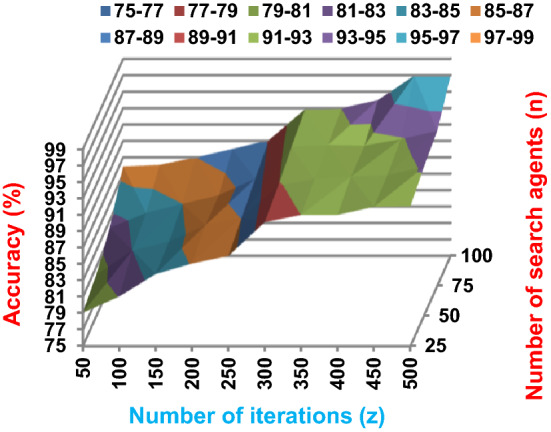
The accuracy of BRPO with collision at n_ck_ = 3

**Fig. 4:**
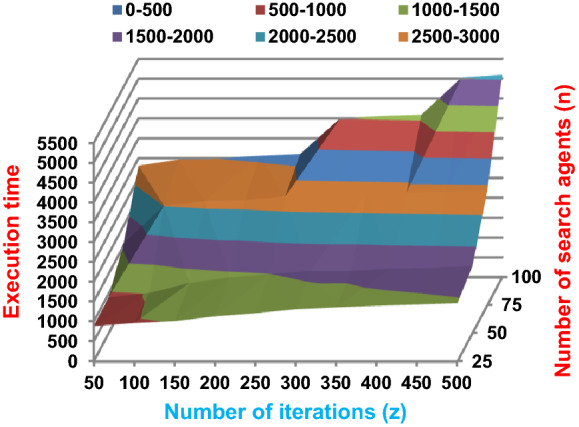
The speed of BRPO with collision at n_ck_ = 3

**Fig. 5:**
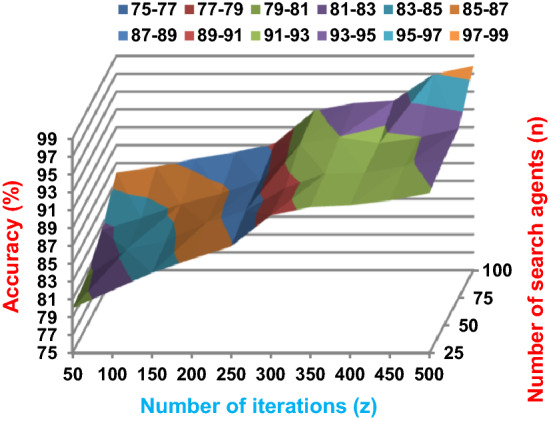
The accuracy of BRPO with collision at n_ck_ = 7

**Fig. 6:**
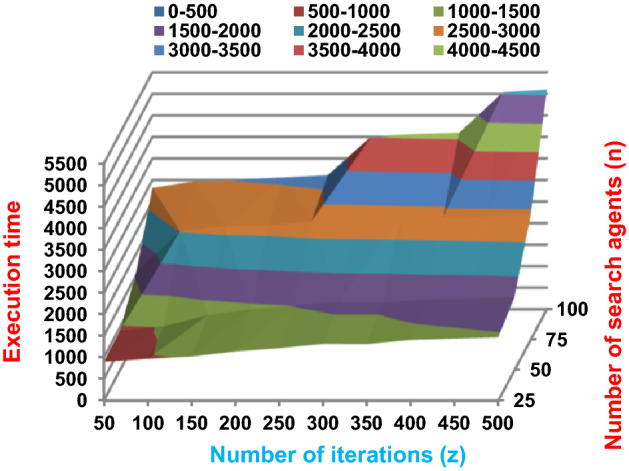
The speed of BRPO with collision at n_ck_ = 7

**Fig. 7:**
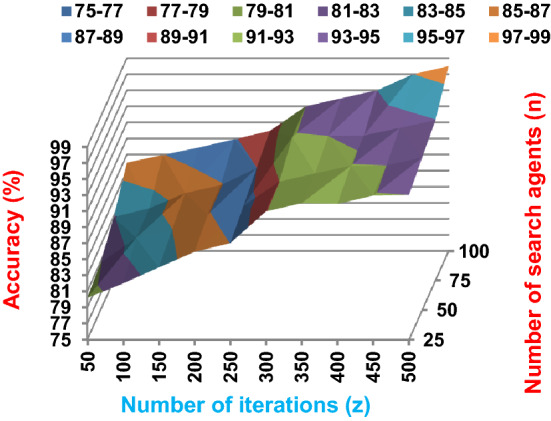
The accuracy of BRPO with collision at n_ck_ = 9

**Fig. 8:**
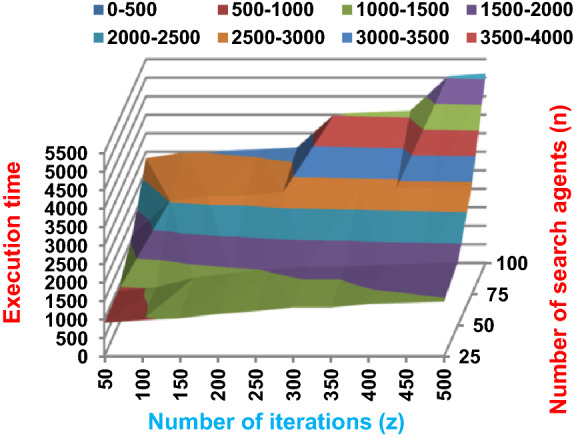
The speed of BRPO with collision at n_ck_ = 9

The BRPO algorithm is implemented in two states; which are, “without collision” and “with collision” according to the collision detection. In each state, two main evaluation metrics called accuracy and execution time, which are presented in (19) and (20), are measured to evaluate the performance of BRPO. In the first state called without collision, the collision between fishes is neglected and the accuracy and execution time of BRPO according to many numbers of iterations (z) and many numbers of search agents (n) are calculated as shown in Figs. 1 and 2 respectively. In the second state called with collision, the collision between fishes is taken in the account and the accuracy and speed of BRPO according to many numbers of iterations (z) and many numbers of search agents (n) are calculated. In the second state, many numbers of checkpoints (n_ck_) which are 3, 7, and 9 are used. Based on these numbers of checkpoints, the BRPO is executed and the accuracy, execution time, micro average precision, micro average recall, macro average precision, macro average recall, and f-measure measurements according to each number of checkpoints are calculated as shown in Figs. 3, 4, 5, 6, 7, 8.

It was mentioned above that Figs. 1 and 2 represent the efficiency of BRPO algorithm in the without collision state. Accordingly, it is noted that Figs. 1 and 2 show that the accuracy and execution time of BRPO algorithm increase gradually with the increase in both the number of iterations (z) and the number of search agents (n). In fact, increasing the execution time of BRPO will be by a small percentage, and this percentage is logical, since by increasing the number of search agents or the number of iterations, the execution time of the algorithm will increase. It is noted that the maximum accuracy values are achieved at number of iterations equals 500 (z = 500), which are; 92%, 93.09%, 93.88%, and 96.99% at number of search agents equals 25, 50, 75, and 100 respectively. Additionally, the minimum execution time values are generated at number of iterations equals 50 (z = 50), which are; 880, 900, 1550, and 2800 at number of search agents equals 25, 50, 75, and 100 respectively. Hence, the maximum accuracy value (96.99%) is provided at 100 search agents (n = 100) and 500 iterations (z = 500) while the minimum execution time value (880) is provided at 25 search agents (n = 25) and 50 iterations (z = 50). It was mentioned above that Figs. 3, 4, 5, 6, 7, 8 represent the efficiency of BRPO algorithm in the with collision state. In Figs. 3, 4, 5, 6, 7, 8, the accuracy and execution time of BRPO algorithm are calculated according to both the number of iterations (z) and the number of search agents (n) using several checkpoints values (n_ck_ = 3, 7, and 9). In fact, Figs. 3 and 4 present the accuracy and execution time of BRPO respectively at n_ck_ = 3, Figs. 5 and 6 present the accuracy and execution time of BRPO respectively at n_ck_ = 7, and Figs. 7 and 8 present the accuracy and execution time of BRPO respectively at n_ck_ = 9. Based on Figs. 3, 4, 5, 6, 7, 8, it is noted that the accuracy and execution time of BRPO algorithm increase gradually with the increase in the number of n_ck_, the number of iterations, and the number of search agents. In the with collision state, the maximum accuracy of BRPO is given at each n_ck_ at 500 iterations (z = 500) and 100 search agents (n = 100) while the minimum execution time of BRPO is given at each n_ck_ is provided at 50 iterations (z = 50) and 25 search agents (n = 25), just like the without collision state. Based on the checkpoints values (n_ck_ = 3, 7, and 9), it is observed that n_ck_ = 3 provides the minimum (worst) accuracy values but the minimum (best) execution time values compared to n_ck_ = 9 and vice versa.

At n_ck_ = 9, the maximum (best) accuracy values are generated at number of iterations equals 500 (z = 500), which are; 93%, 93.99%, 94.95%, and 98% at number of search agents equals 25, 50, 75, and 100 respectively. Also, the minimum (best) execution time values are introduced at number of iterations equals 50 (z = 50), which are; 911, 929, 1580, and 2828 at number of search agents equals 25, 50, 75, and 100 respectively. Hence, at n_ck_ = 9, the maximum accuracy value (98%) is provided at 100 search agents (n = 100) and 500 iterations (z = 500) while the minimum execution value (911) is provided at 25 search agents (n = 25) and 50 iterations (z = 50). Based on these results, it is observed that the increase of execution time in "with collision state" is a very a slight increase, not large, in order to be noticeable and affect the performance of the BRPO algorithm. Compared to the without collision state, it is concluded that the accuracy of the BRPO algorithm is lower than "with collision state" and its execution time is slightly lower than the with collision state. Thus, the BRPO algorithm based on "with collision state" cannot be suitable for real time applications due to the increased execution time. In the next subsection, a comparative study between the BRPO algorithm in both states (without collision and with collision at n_ck_ = 9) and the other nine algorithms provided in Table 7 will be introduced according to the number of iterations (z). This study will be performed based on each number of search agents (25, 50, 75, and 100) separately to measure the performance of BRPO algorithm against others.

b. **Comparing the Performance of BRPO with Related Competitors**

**Fig. 9:**
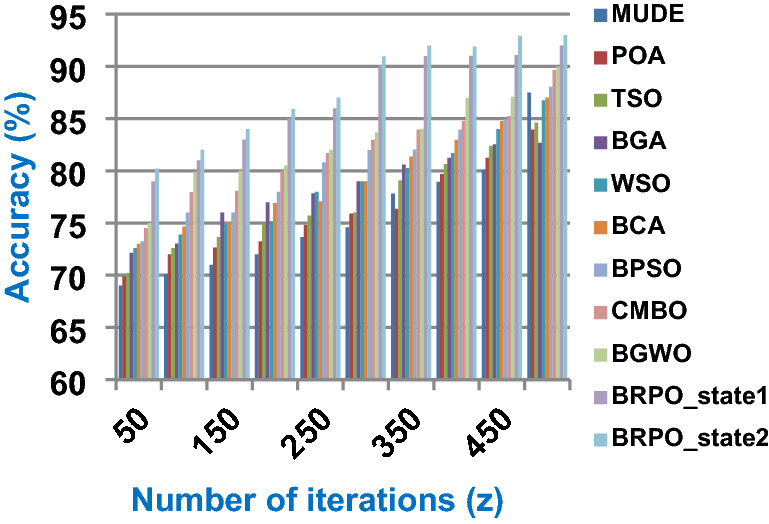
The accuracy of the proposed BRPO and the other competitors when search agents (n = 25)

**Fig. 10:**
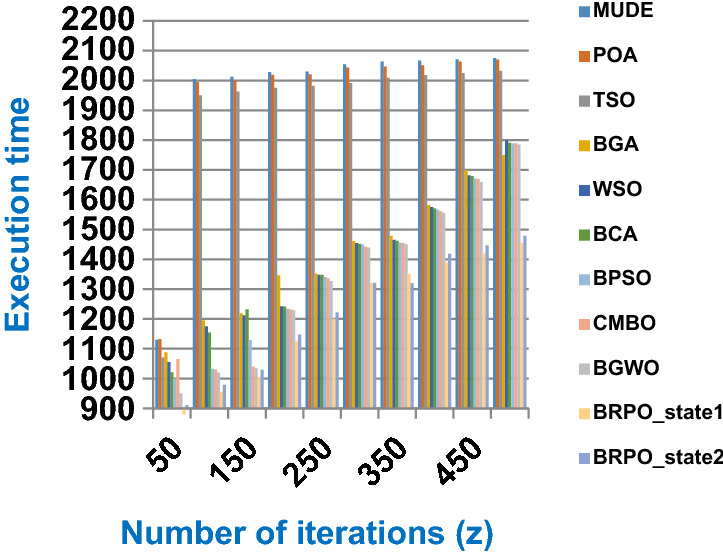
The execution time of the proposed BRPO and the other competitors when search agents (n = 25)

**Fig. 11:**
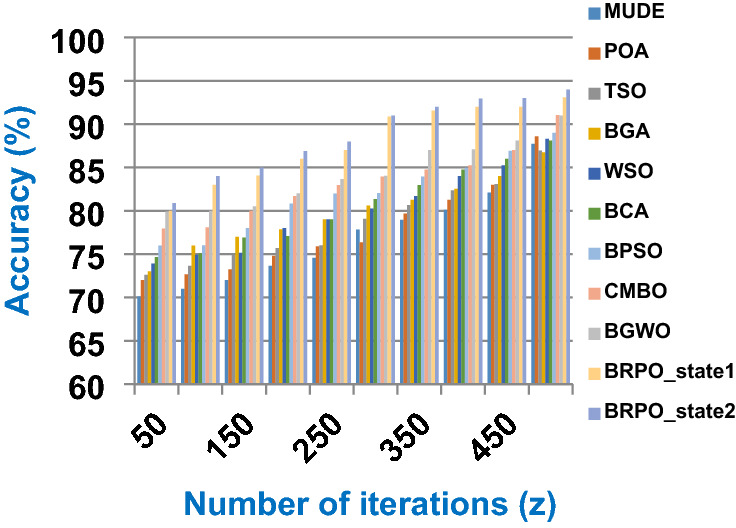
The accuracy of the proposed BRPO and the other competitors when search agents (n = 50)

**Fig. 12:**
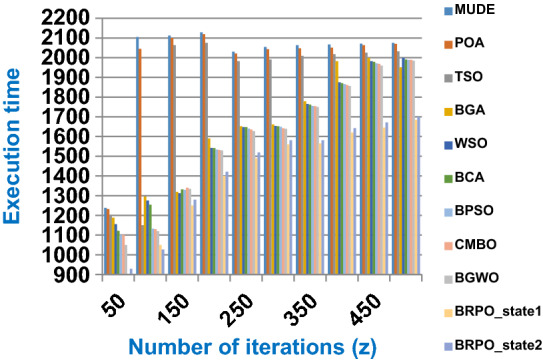
The execution time of the proposed BRPO and the other competitors when search agents (n = 50)

**Fig. 13:**
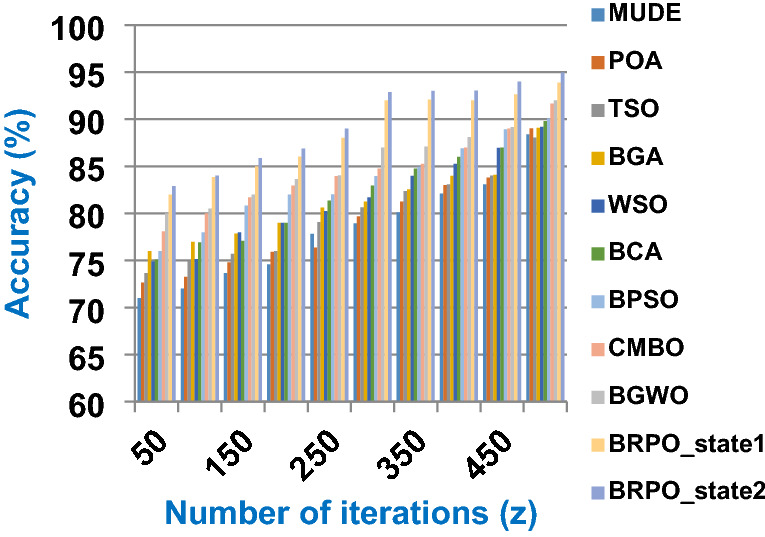
The accuracy of the proposed BRPO and the other competitors when search agents (n = 75)

**Fig. 14:**
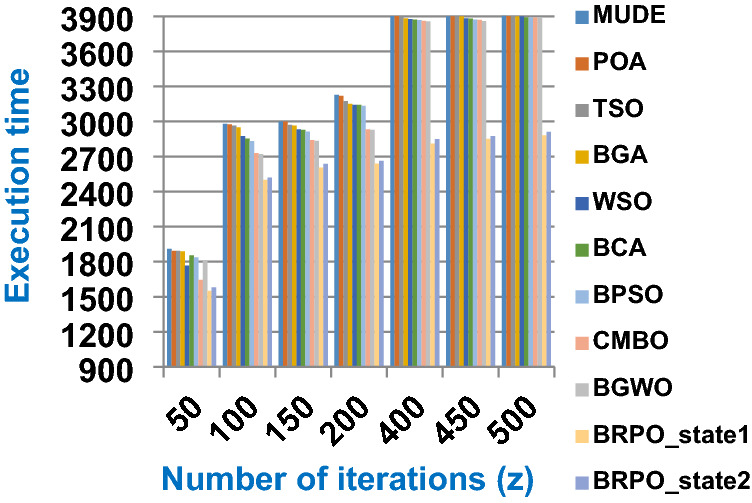
The execution time of the proposed BRPO and the other competitors when search agents (n = 75)

**Fig. 15:**
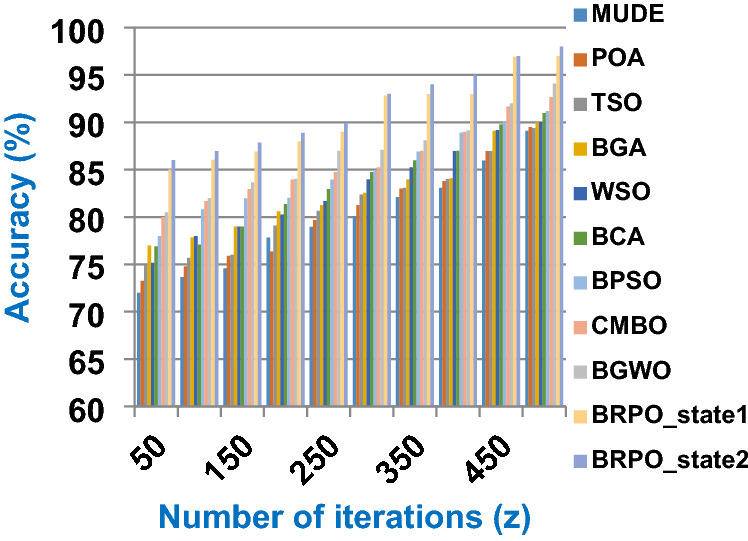
The accuracy of the proposed BRPO and the other competitors when search agents (n = 100)

**Fig. 16:**
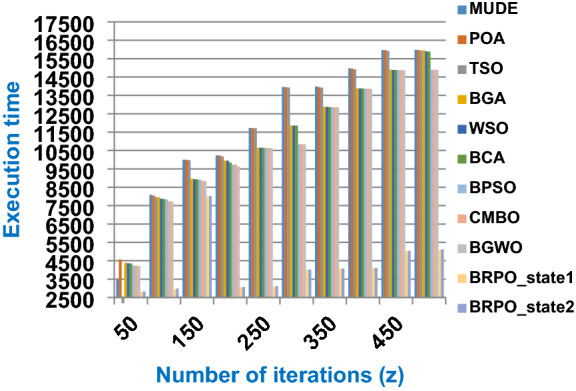
The execution time of the proposed BRPO and the other competitors when search agents (n = 100)

**Fig. 17:**
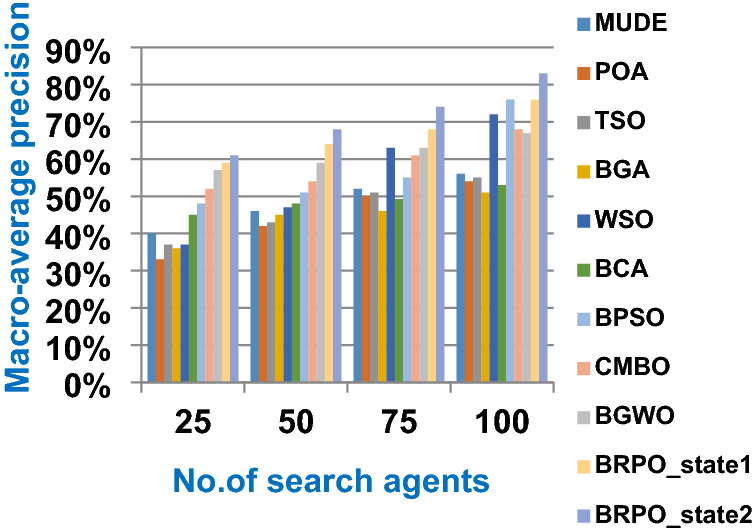
Macro average precision of the proposed BRPO and the other competitors

**Fig. 18:**
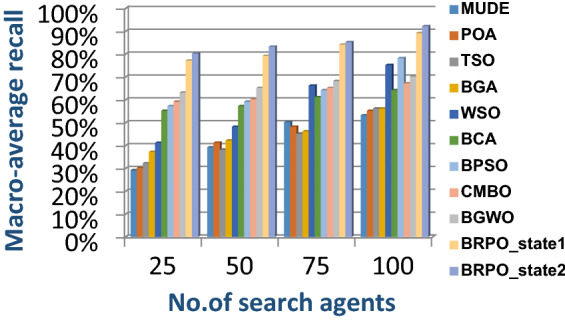
Macro average recall of the proposed BRPO and the other competitors

**Fig. 19:**
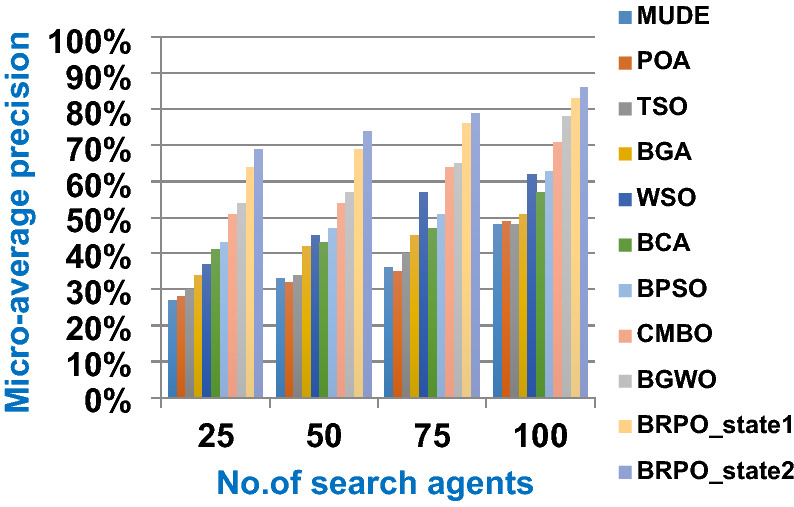
Micro average precision of the proposed BRPO and the other competitors

**Fig. 20:**
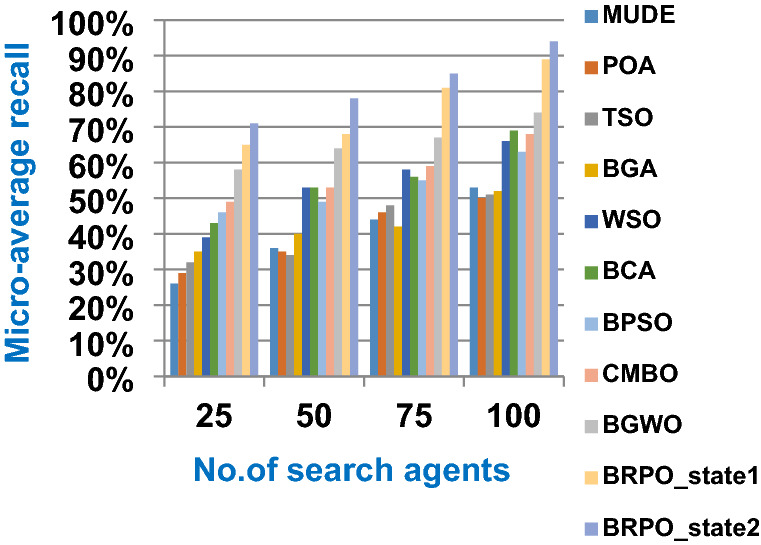
Micro average recall of the proposed BRPO and the other competitors

**Fig. 21:**
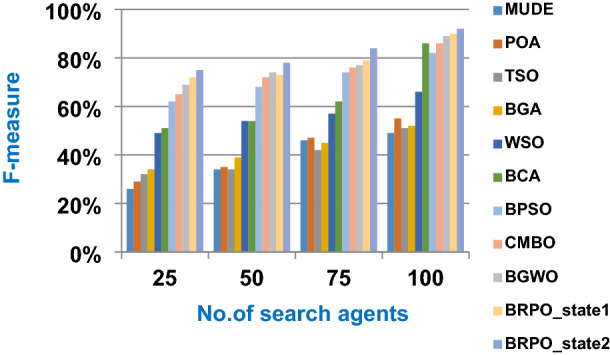
F-measure of the proposed BRPO and the other competitors

In this comparative study, the performance of BRPO algorithm is measured to demonstrate its effectiveness against the performance of other nine algorithms presented in Table 7. Practically, accuracy execution time, micro average precision, micro average recall, macro average precision, macro average recall, and f-measure are used as performance metrics to measure the efficiency of BRPO in both states (without collision and with collision at n_ck_ = 9) against other algorithms according to the number of iterations (z). In fact, the execution of BRPO based on the without collision is denoted as “BRPO_state1” while its execution based on "with collision state" is denoted as “BRPO_state2”. These measurements are performed according to each number of search agents (25, 50, 75, and 100) separately. Based on these numbers of search agents, the BRPO is executed and the accuracy, execution time, micro average precision, micro average recall, macro average precision, macro average recall, and f-measure measurements according to each number of search agents (n) separately are calculated as shown in Figs. 9, 10, 11, 12, 13, 14, 15, 16, 17, 18, 19, 20, 21.

As presented in Figs. 9, 10, 11, 12, 13, 14, 15, 16, the accuracy and execution time of all algorithms increase gradually with the increase in the number of iterations (z) and also with the increase in the number of search agents (n). Based on 25 search agents (n = 25), Fig. 9 shows the accuracy of all algorithms while Fig. 10 provides their execution time according to many numbers of the iterations (z) presented in Table 8. In Fig. 9, it is noted that the BRPO_state2 provides the maximum (best) accuracy value but the BGA provides the minimum (worst) accuracy value according to all numbers of iterations. The best (maximum) accuracy values of MUDE, POA, TSO, BGA, WSO, BCA, BPSO, CMBO, BGWO, BRPO_state1, and BRPO_state2 are 87.5%, 88.01%, 84.82%, 82.67%, 86.754%, 87.02%, 88.05%, 89.4.65%, 89.4.99%, 92%, and 93% respectively at the maximum iterations number (z = 500). In Fig. 10, it is noted that the BRPO_state1 provides the minimum execution time value according to all numbers of iterations. The best (minimum) execution time values of MUDE, POA, TSO, BGA, WSO, BCA, BPSO, CMBO, BGWO, BRPO_state1, and BRPO_state2 are 1130, 1160, 1070, 1088, 1055, 1021, 1065, 1065, 950, 880, and 911 respectively at the minimum iterations number (z = 50). It is noted that the best accuracy values of all algorithms are given at the maximum iterations number (z = 500) while their best execution time values are provided at the minimum iterations number (z = 50). Thus, BRPO outperforms other algorithms at n = 25 where BRPO_state2 can provide the maximum accuracy value and BRPO_state1 can provide the minimum execution value at z = 500 compared to the other algorithms. Based on 50 search agents (n = 50), the accuracy values of all algorithms are provided in Fig. 11 and their execution time are introduced in Fig. 12. At n = 50, the accuracy and execution time of all algorithms increase more than the values at n = 25. Figure 11 shows that the maximum accuracy values are provided by the BRPO_state2 while the minimum values are introduced by the BGA through all numbers of iterations with values reach to 93.99% and 86.754% respectively at z = 500. At z = 500, the maximum accuracy values of MUDE, POA, TSO, BGA, WSO, BCA, BPSO, CMBO, BGWO, BRPO_state1, and BRPO_state2 are 87.72%, 88.6%, 86.754%, 86.932%,, 88.301%, 88.1%, 88.99%, 91.03%, 90.99%, 93.09%, and 93.99% respectively. As shown in Fig. 12, the minimum execution time values are provided by the BRPO_state1 and the maximum values are provided by the MUDE based on all numbers of iterations with values reach to 900 and 1238 respectively at the maximum iteration number (z = 500). The minimum execution time values of MUDE, POA, TSO, BGA, WSO, BCA, BPSO, CMBO, BGWO, BRPO_state1, and BRPO_state2 are 1238, 1232, 1202, 1188, 1155, 1121, 1100, 1100, 1050, 900, and 929 respectively at z = 50. According to all algorithms, the best accuracy values are provided when z = 500 but their best execution time values are provided when z = 50. When n = 500, the maximum accuracy and minimum execution time values are given by the BRPO_state2 and the BRPO_state1 respectively compared to the other algorithms. When the number of search agents is 75 (n = 75), Figs. 13 and 14 show the accuracy and execution time values respectively for all algorithms. The accuracy and execution time of all algorithms at n = 75 increase more than the values at n = 25 or n = 50.Through all numbers of iterations, Fig. 13 provides that the BRPO_state2 gives the maximum accuracy values but the TSO gives the minimum values. When z = 500, the accuracy of BRPO_state2 is 94.95% while the accuracy of the TSO is 88.04%. The maximum accuracy values of MUDE, POA, TSO, BGA, WSO, BCA, BPSO, CMBO, BGWO, BRPO_state1, and BRPO_state2 are provided at z = 500 with values equal 88.4%, 89.4,88.04%, 87.92%, 86.754%, 89.4.09%, 89.4.19%, 89.4.79%, 90.22%, 91.65%, 93.88%, and 94.95% respectively. On the other hand, Fig. 14 shows that the minimum execution time values are given by the BRPO_state1 while the maximum values are given by the MUDE according to all numbers of iterations with values reach to 1550 and 1910 respectively at z = 500. All algorithms provide the minimum execution time values at z = 50 where the execution time of MUDE, POA, TSO, BGA, WSO, BCA, BPSO, CMBO, BGWO, BRPO_state1, and BRPO_state2 are 1910, 1892, 1829, 1888, 1767, 1767, 1836, 1645, 1814, 1550, and 1580 respectively. Hence, at the minimum iterations number (z = 50), all algorithms provide their best execution time values but their best accuracy values are given at the maximum iterations number (z = 500). Comparing the BRPO algorithm with other algorithms, it is concluded that the maximum accuracy values are given by the BRPO_state2 while the minimum execution time values are provided by the BRPO_state1.When the number of search agents is 100 (n = 100), the accuracy and execution time of all algorithms are provided in Figs. 15 and 16 respectively. At n = 100, the accuracy and execution time of all algorithms increase more than the values at n = 25, n = 50, or n = 75. In Fig. 15, the best accuracy values are introduced by the BRPO_state2 while the worst values are given by the MUDE where the accuracy of BRPO_state2 is 98% while the accuracy of the MUDE is 89.1% when z = 500. When z = 500, the maximum accuracy values of MUDE, POA, TSO, BGA, WSO, BCA, BPSO, CMBO, BGWO, BRPO_state1, and BRPO_state2 are provided with values equal 89.1%, 89.4.5%, 89.4%, 89.4.99%, 90.01%, 90.98%, 91.19%, 92.65%, 94.09%, 96.99%, and 98% respectively. According to Fig. 16, the best execution time values are given by the BRPO_state1 while the worst values are provided by the MUDE through all numbers of iterations with values reach to 2800 and 3460 respectively at z = 500. The minimum execution time values of all algorithms are introduced at z = 50 where the execution time of MUDE, POA, TSO, BGA, WSO, BCA, BPSO, CMBO, BGWO, BRPO_state1, and BRPO_state2 are 3460, 4569, 2190, 4382, 4355, 4345, 4236, 4221, 4214, 2800, and 2828 respectively. Accordingly, all algorithms provide their best accuracy values at z = 500 while their best execution time values are given at z = 50. According to Figs. 9, 10, 11, 12, 13, 14, 15, 16, it is concluded that the maximum accuracy values are given by the BRPO_state2 while the minimum execution time values are provided by the BRPO_state1 through all numbers of iterations. Thus, the BRPO has proven its effectiveness against other algorithms in terms of accuracy and execution time. Tables 9, 10, 11, 12, 13, 14, 15, 16, 17, 18, 19, 20) show the summary of results for the accuracy and execution time of all algorithms.

**Table 9:** Accuracy and execution time of RPO (without collision)

		50		100		150		200		250		300		350		400		450		500	
		Accuracy	Time	Accuracy	Time	Accuracy	Time	Accuracy	Time	Accuracy	Time	Accuracy	Time	Accuracy	Time	Accuracy	Time	Accuracy	Time	Accuracy	Time
Number of search agents (n)	25	79	880	81	955	83	1005	85	1125	85.99	1200	90.1	1320	90.99	1350	91	1390	91.08	1420	92	1455
	50	80	900	83	1050	84.05	1238	86	1400	87	1490	90.88	1560	91.55	1565	91.99	1620	92	1645	93.09	1685
	75	82	1550	83.85	2500	84.99	2604	86.03	2640	88.02	2731	91.99	2761	92.08	2795	92	2810	92.65	2850	93.88	2880
	100	85	2800	86.03	2950	86.91	3002	88	3048	89.4	3090	92.82	4001	92.99	4050	92.99	4089.4	96.9	5020	96.99	5082

**Table 10:** Accuracy and execution time of RPO (with collision &number of checks = 3)

		50		100		150		200		250		300		350		400		450		500	
		Accuracy	Time	Accuracy	Time	Accuracy	Time	Accuracy	Time	Accuracy	Time	Accuracy	Time	Accuracy	Time	Accuracy	Time	Accuracy	Time	Accuracy	Time
Number of search agents (n)	25	79.1	89.40	81.06	965	83.8	1015	85.09	1135	86.01	1212	90.13	1313	91.01	1362	91.02	1402	91.99	1432	92.05	1466
	50	80.05	910	83.02	1015	84.09	1270	86.08	1410	87.05	1500	90.9	1572	91.69	1570	92	1632	92.19	1655	93.15	1691
	75	82.09	1560	83.89.4	2510	85	2620	86.07	2650	88.08	2741	92	2774	92.18	2805	92.09	2836	92.89.4	2862	93.91	289.43
	100	85.9	2810	86.09	2965	87	3010	88.03	3058	89.4.09	3105	92.88	4014	93.01	4062	93.99	4095	96.95	5036	97.01	5097

**Table 11:** Accuracy and execution time of RPO (with collision &number of checks = 7)

		50		100		150		200		250		300		350		400		450		500	
		Accuracy	Time	Accuracy	Time	Accuracy	Time	Accuracy	Time	Accuracy	Time	Accuracy	Time	Accuracy	Time	Accuracy	Time	Accuracy	Time	Accuracy	Time
Number of search agents (n)	25	79.99	905	81.99	971	88.01	1025	85.49	1141	86.91	1218	90.39	1317	91.31	1382	91.52	1409	92.05	1440	92.9	1471
	50	80.55	923	87.5	1020	84.5	1276	86.48	1418	87.85	1512	90.41	1577	91.99	1577	92.12	1639	92.79	1665	93.59	1699
	75	82.59	1574	88.01	2517	85.5	2628	82.67	2659	88.17	2748	92.25	2777	92.98	2810	92.29	2840	92.94	2869	93.97	2902
	100	85.95	2820	86.59	2975	87.51	3019	88.23	3062	89.4.21	3114	92.91	4019	93.71	4069	94.01	4100	96.96	5040	97.9	5100

**Table 12:** Accuracy and execution time of RPO (with collision &number of checks = 9)

		50		100		150		200		250		300		350		400		450		500	
		Accuracy	Time	Accuracy	Time	Accuracy	Time	Accuracy	Time	Accuracy	Time	Accuracy	Time	Accuracy	Time	Accuracy	Time	Accuracy	Time	Accuracy	Time
Number of search agents (n)	25	80.2	911	82	979	83.99	1029	85.93	1147	87.02	1222	90.96	1321	91.99	1320	91.9	1418	92.92	1447	93	1479
	50	80.89.4	929	83.99	1027	84.98	1279	86.89.4	1421	87.99	1519	90.99	1581	92	1581	92.95	1643	93	1671	93.99	1700
	75	82.9	1580	84	2520	85.88	2638	86.89.4	2663	88.99	2752	92.89.4	2782	93	2819	93.05	2849	94	2874	94.95	2910
	100	86	2828	89.4.04	2985	87.87	3029	88.9	3069	89.4.95	3120	93	4025	94.01	4074	95	4110	96.99	5049	98	5115

**Table 13:** The accuracy of the proposed BRPO and the other competitors when search agents (n = 25)

Number of search agent = 25		Evaluation function calls FE's									
		50	100	150	200	250	300	350	400	450	500
Optimization algorithm	MUDE	69	70.1	70.99	72	73.65	74.58	77.82	78.95	80.1	87.5
	POA	69.9	71.99	72.65	73.25	74.8	75.9	76.35	79.68	81.25	88.01
	TSO	70.2	72.59	73.65	74.92	75.7	76	79.08	80.65	82.36	84.82
	BGA	72.15	73.02	75.99	76.98	77.85	79	80.6	81.25	82.54	82.67
	WSO	72.59	73.9	74.9	75.15	77.99	79	80.25	81.69	83.99	86.75
	BCA	73	74.65	75.08	76.91	77.08	79	81.36	82.96	84.75	87.02
	BPSO	73.25	75.99	76	77.99	80.82	81.98	82.03	88.01	84.95	88.05
	CMBO	74.5	77.95	78.09	80	81.69	82.96	88.01	84.75	85.25	89.4.6
	BGWO	75	79.8	80	80.5	82	83.65	84.025	86.754	88.30	89.4.9
	BRPO_state1	79	81	83	85	85.99	90.1	90.99	91	91.08	92
	BRPO_state2	80.2	82	83.99	85.93	87.02	90.96	91.99	91.9	92.92	93

**Table 14:** The execution time of the proposed BRPO and the other competitors when search agents (n = 25)

Number of search agent = 25		Evaluation function calls FE's									
		50	100	150	200	250	300	350	400	450	500
**Optimization algorithm**	MUDE	1130	2005	2012	2028	2030	2054	2063	2067	2071	2075
	POA	1160	1995	2000	2019	2021	2043	2047	2051	2063	2070
	TSO	1070	1950	1963	1975	1982	1991	2009	2018	2025	2032
	BGA	1088	1195	1219	1346	1352	1461	1479	1582	1699	1750
	WSO	1055	1175	1212	1242	1348	1454	1465	1575	1682	1799
	BCA	1021	1154	1232	1241	1348	1452	1462	1571	1679	1791
	BPSO	1065	1032	1129	1234	1340	1449	1455	1566	1671	1789.4
	CMBO	1065	1030	1040	1232	1336	1442	1454	1561	1669	1789.4
	BGWO	950	1020	1034	1229	1327	1439	1450	1556	1659	1785
	BRPO_state1	880	955	1005	1125	1200	1320	1350	1390	1420	1455
	BRPO_state2	911	979	1029	1147	1222	1321	1320	1418	1447	1479

**Table 15:** The accuracy of the proposed BRPO and the other competitors when search agents (n = 50)

Number of search agent = 50		Evaluation function calls FE's									
		50	100	150	200	250	300	350	400	450	500
Optimization algorithm	MUDE	70.1	70.99	72	73.65	74.58	77.82	78.95	80.1	82.1	87.72
	POA	71.99	72.65	73.25	74.8	75.9	76.35	79.68	81.25	82.99	88.6
	TSO	72.59	73.65	74.92	75.7	76	79.08	80.65	82.36	83.09	88.932
	BGA	73.02	75.99	76.98	77.85	79	80.6	81.25	82.54	83.99	86.754
	WSO	73.9	74.9	75.15	77.99	79	80.25	81.69	83.99	85.25	88.301
	BCA	74.65	75.08	76.91	77.08	79	81.36	82.96	84.75	85.99	88.1
	BPSO	75.99	76	77.99	80.82	81.98	82.03	88.01	84.95	86.91	88.99
	CMBO	77.95	78.09	80	81.69	82.96	88.01	84.75	85.25	86.754	90
	BGWO	79.89.4	80	80.5	82	83.65	84.025	86.754	88.301	89.4.5	90.99
	BRPO_state1	80	83	84.05	86	87	90.88	91.55	91.99	92	93.09
	BRPO_state2	80.89	83.99	84.98	86.89	87.99	90.99	92	92.95	93	93.99

**Table 16:** The execution time of the proposed BRPO and the other competitors when search agents (n = 50)

Number of search agent = 50		Evaluation function calls FE's									
		50	100	150	200	250	300	350	400	450	500
Optimization algorithm	MUDE	1238	2105	2112	2128	2030	2054	2063	2067	2071	2075
	POA	1232	2045	2100	2119	2021	2043	2047	2051	2063	2070
	TSO	1202	1130	2063	2075	1982	1991	2009	2018	2025	2032
	BGA	1188	1295	1319	1590	1652	1661	1779	1982	1999	1951
	WSO	1155	1275	1312	1542	1648	1654	1765	1875	1982	1999
	BCA	1121	1254	1332	1541	1648	1652	1762	1871	1979	1991
	BPSO	1100	1160	1329	1534	1640	1649	1755	1866	1971	1989
	CMBO	1100	1130	1340	1532	1636	1642	1754	1861	1969	1989
	BGWO	1050	1120	1334	1529	1627	1639	1750	1856	1959	1985
	BRPO_state1	900	1050	1238	1400	1490	1560	1565	1620	1645	1685
	BRPO_state2	929	1027	1279	1421	1519	1581	1581	1643	1671	1700

**Table 17:** The accuracy of the proposed BRPO and the other competitors when search agents (n = 75)

Number of search agent = 75		Evaluation function calls FE's					
		50	100	150	400	450	500
Optimization algorithm	MUDE	70.99	72	73.65	82.1	83.09	88.4
	POA	72.65	73.25	74.8	82.99	83.79	89.4.04
	TSO	73.65	74.92	75.7	83.09	84	89.4.04
	BGA	75.99	76.98	77.85	83.99	84.09	89.4.09
	WSO	74.9	75.15	77.99	85.25	89.4.04	89.4.19
	BCA	75.08	76.91	77.08	85.99	86.754	89.4.79
	BPSO	76	77.99	80.82	86.91	88.91	90.22
	CMBO	78.09	80	81.69	86.754	88.99	91.65
	BGWO	80	80.5	82	89.4.5	89.4.15	91.99
	BRPO_state1	82	83.85	84.99	92	92.65	93.88
	BRPO_state2	82.9	84	85.88	93.05	94	94.95

**Table 18:** The execution time of the proposed BRPO and the other competitors when search agents (n = 75)

Number of search agent = 75		Evaluation function calls FE's					
		50	100	150	400	450	500
Optimization algorithm	MUDE	1910	2980	2998	3967	3971	3975
	POA	189.42	2975	2998	3951	3963	3970
	TSO	189.42	2965	2970	3918	3925	3932
	BGA	1888	2950	2965	3882	389.49	3951
	WSO	1767	2875	2932	3875	3882	389.49
	BCA	1767	2854	2929	3871	3879	389.41
	BPSO	1836	2832	2912	3866	3871	3889.4
	CMBO	1645	2730	2840	3861	3869	3889.4
	BGWO	1814	2720	2834	3856	3859	3885
	BRPO_state1	1550	2500	2604	2810	2850	2880
	BRPO_state2	1580	2520	2638	2849	2874	2910

**Table 19:** The accuracy of the proposed BRPO and the other competitors when search agents (n = 100)

Number of search agent = 100		Evaluation function calls FE's					
		50	100	150	400	450	500
Optimization algorithm	MUDE	72	73.65	74.58	83.09	88.4	89.4
	POA	73.25	74.8	75.9	83.79	89.4.04	89.4
	TSO	74.92	75.7	76	84	89.4.04	89.4
	BGA	76.98	77.85	79	84.09	89.4.09	89.
	WSO	75.15	77.99	79	89.4.04	89.4.19	90.01
	BCA	76.91	77.08	79	86.754	89.4.79	90.98
	BPSO	77.99	80.82	81.98	88.91	90.22	91.19
	CMBO	80	81.69	82.96	88.99	91.65	92.65
	BGWO	80.5	82	83.65	89.4.15	91.99	94.09
	BRPO_state1	85	86.03	86.91	92.99	96.9	96.99
	BRPO_state2	86	89.4.04	87.87	95	96.99	98

**Table 20:** The execution time of the proposed BRPO and the other competitors when search agents (n = 100)

Number of search agent = 100		Evaluation function calls FE's					
		50	100	150	400	450	500
Optimization algorithm	MUDE	3460	8080	9998	14,967	15,971	15,975
	POA	4569	8050	9998	14,951	15,963	15,970
	TSO	2190	7965	9970	14,918	15,925	15,932
	BGA	4382	7950	89.465	13,882	1489.49	15,951
	WSO	4355	7875	89.432	13,875	14,882	1589.49
	BCA	4345	7854	89.429	13,871	14,879	1589.41
	BPSO	4236	7832	89.412	13,866	14,871	14,889.4
	CMBO	4221	7730	8840	13,861	14,869	14,889.4
	BGWO	4214	7720	8834	13,856	14,859	14,885
	BRPO_state1	2800	2950	8002	4089.4	5020	5082
	BRPO_state2	2828	2985	8029	4110	5049	5115

The results in Figs. 17, 18, 19, 20, 21 show that the highest macro-average precision value is provided by BRPO state_2 with a value that reaches 0.83 at number of search agent equals to 100. On the other hand, the lowest macro-average precision value is introduced by BGA with a value reaches to 0.51. Additionally, macro-average recall for BRPO_ state2 is about 0.92 which represents the highest value concerning other algorithms, while the lowest one is BGA with a value of 0.53 at number of search agents equals 100. At 100 search agents, BRPO state _2 gives the highest micro-average precision value equals 0.86 while TSO introduced 0.49 which is the lowest value of micro-average precision. BRPO state_2 provides a micro average recall value that equals 0.94. F-measure value for BRPO is about 0.92 while it is about 0.49, 0.55, 0.51, 0.52, 0.66, 0.86, 0.82, 0.86, 0.89, and 0.90 for MUDE, POA, TSO, BGA, WSO, BCA, BPSO, CMBO, BGWO, BRBO_state1 respectively.

## Conclusions and future work

Online applications need to fast and accurate optimization algorithms. Thus, researchers seek to provide the fastest and the most accurate optimization algorithm. The accuracy and execution time of the conventional optimization algorithms increase gradually with the increase in the number of search agents and the number of iterations. Thus, a new optimization algorithm called Red Piranha Optimization (RPO) algorithm is introduced in this paper to provide the best accuracy and execution time compared to the other nine algorithms according to many numbers of search agents and many numbers of iterations. In fact, the RPO algorithm is evaluated according to two states called without collision and with collision states. Feature selection using Binary RPO (BRPO) algorithm is implemented in this work as a case study to evaluate the effectiveness of BRPO against the other nine algorithms in their binary form to quickly and accurately select the best subset of features from the Albert Einstein dataset that contains 110 features. The experimental work is performed for many numbers of iterations (50, 100, 150, 200, 250, 300, 350, 400, 450, 500) and also for many numbers of search agents (25, 50, 75, 100) for all optimization algorithms. The maximum accuracy values for all optimization algorithms are provided at the number of iterations equals 500 and the number of search agent equals 100. On the other hand, the minimum execution time values for all optimization algorithms are provided at the number of iterations equals 50 and the number of search agents equals 25. Compared to the conventional nine algorithms, the BRPO algorithm gives the maximum accuracy values but gives the minimum execution time values according to all numbers of iterations and all numbers of search agents. The maximum accuracy values are introduced by BRPO based on the with collision state while the minimum execution time values are given by BRPO based on the without collision state. Also, the BRPO outperforms the other nine algorithms according to micro average precision, micro average recall, macro average precision, macro average recall, and f-measure calculations.
